# Transcriptional profiling of chickpea genes differentially regulated in response to high-salinity, cold and drought

**DOI:** 10.1186/1471-2164-8-303

**Published:** 2007-09-02

**Authors:** Nitin L Mantri, Rebecca Ford, Tristan E Coram, Edwin CK Pang

**Affiliations:** 1 RMIT University, School of Applied Sciences, Biotechnology and Environmental Biology, Building 223, Level 1, Plenty Road, Bundoora, Victoria. 3083. Australia; 2 BioMarka, Faculty of Land and Food Resources, The University of Melbourne, Victoria. 3010. Australia; 3 United States Department of Agriculture, Agricultural Research Service, Wheat Genetics, Quality, Physiology and Disease Research Unit and Department of Plant Pathology, Washington State University, Pullman, WA, 99164-6430. USA

## Abstract

**Background:**

Cultivated chickpea (*Cicer arietinum*) has a narrow genetic base making it difficult for breeders to produce new elite cultivars with durable resistance to major biotic and abiotic stresses. As an alternative to genome mapping, microarrays have recently been applied in crop species to identify and assess the function of putative genes thought to be involved in plant abiotic stress and defence responses. In the present study, a cDNA microarray approach was taken in order to determine if the transcription of genes, from a set of previously identified putative stress-responsive genes from chickpea and its close relative *Lathyrus sativus*, were altered in chickpea by the three abiotic stresses; drought, cold and high-salinity. For this, chickpea genotypes known to be tolerant and susceptible to each abiotic stress were challenged and gene expression in the leaf, root and/or flower tissues was studied. The transcripts that were differentially expressed among stressed and unstressed plants in response to the particular stress were analysed in the context of tolerant/susceptible genotypes.

**Results:**

The transcriptional change of more than two fold was observed for 109, 210 and 386 genes after drought, cold and high-salinity treatments, respectively. Among these, two, 15 and 30 genes were consensually differentially expressed (DE) between tolerant and susceptible genotypes studied for drought, cold and high-salinity, respectively. The genes that were DE in tolerant and susceptible genotypes under abiotic stresses code for various functional and regulatory proteins. Significant differences in stress responses were observed within and between tolerant and susceptible genotypes highlighting the multiple gene control and complexity of abiotic stress response mechanism in chickpea.

**Conclusion:**

The annotation of these genes suggests that they may have a role in abiotic stress response and are potential candidates for tolerance/susceptibility.

## Background

Chickpea (*Cicer arietinum *L.) is an annual, self-pollinating, diploid (2n = 2x = 16) pulse crop that ranks third in world legume production [1]. Australia is currently the largest exporter of chickpea and yield for 2006–2007 has been forecasted at 239,000 tonnes, most of which will be exported (225,000 tonnes) [2]. Under optimum growing conditions, the yield potential of chickpea is 6 t/ha [3], which is much higher than the current global yield average of ~0.8 t/ha [1]. The chief constraints in chickpea production are biotic stresses like *Ascochyta *blight, *Fusarium *wilt, pod borer, and abiotic stresses such as drought, heat, cold and high-salinity [4]. In fact, the estimated collective yield losses due to abiotic stresses (6.4 million t) have been significantly higher than for biotic stresses (4.8 million t) [4]. Drought, a severe abiotic stress of chickpea, causes a 40–50% reduction in yield globally [1]. The change from spring to winter sowing of chickpea for efficient utilization of rain water in Mediterranean environments has enhanced yields, but demands tolerance to low temperature for further yield improvements [5]. Most legumes are known to be salt sensitive [6], and the increasing worldwide use of irrigation has led to the prediction that, by 2050, 50% of all arable land will be salinized [7]. Therefore, it is becoming increasingly important to produce cultivars tolerant to high-salinity in addition to other abiotic and biotic stresses for sustainable chickpea production.

The cultivated chickpea has a high morphological but narrow genetic variation [8], which makes it difficult for breeders to produce elite cultivars with durable resistance to the many major biotic and abiotic stresses. Molecular markers associated with quantitative trait loci (QTL) for resistance to biotic stresses and some morphological traits have been located on both interspecific [9-18] and intraspecific linkage maps [19-22]. However compared to some biotic stresses, abiotic stresses are inherited in a more quantitative manner and may be subjective to assess under field conditions due to confounding environmental factors, which makes it difficult to screen for and quantitate tolerance. Quantitating the effects of abiotic stresses involves measurement of various factors like survival rate, yield, dry matter production, days to maturity, flower/pod survival, root mass and transpiration ratio. Their tolerances are likely to be quantitatively controlled and this feature of abiotic stresses represents a major obstacle to developing molecular markers.

Marker-assisted breeding is increasingly targeted towards tracking the candidate genes responsible for stress tolerance through gene identification and functional studies [23]. Candidate genes, identified and characterized through whole genome sequencing projects or expressed sequence tag (EST) libraries, are assessed for their comparative transcriptional activity against biological reactions to specific plant stresses via microarray technologies. Analysis of the expression and function/s of stress inducible genes facilitates understanding of the molecular mechanisms underlying the stress tolerance responses. This approach has potential to assist molecular plant breeders in improving stress tolerance by gene selection and/or genetic manipulation. Gene expression studies using EST-based cDNA microarrays were pioneered by analysing 48 *Arabidopsis *genes for differential expression in roots and shoots [24]. The technology has subsequently been used extensively to generate expression profiles of genes linked to drought, heat, cold and salt stresses [25-29]. In order to obtain a complete picture of a plant's response to stress, it would be ideal to study the expression profiles of all the genes in its genome. Currently, this is only possible for model crops like *Arabidopsis thaliana *(thale cress), *Oryza sativa *(rice), *Medicago truncatula *(barrel medic), *Populus trichocarpa *(black cottonwood) whose genomes have been sequenced. In near future it shall be possible for *Brachypodium distachyon*, *Lotus japonicus *(lotus), *Manihot esculenta *(cassava), *Solanum lycopersicum *(tomato), *Solanum tuberosum *(potato), *Sorghum bicolor *and *Zea mays *(corn) whose genome sequencing shall be soon completed [30]. Until this is available for other crops, researchers have to rely on information generated by studying these model crops and explore the EST/cDNA sequences from same/related species generated by various studies. For pulses, a set of chickpea unigenes [31], grasspea ESTs [32] and lentil RGA sequences (Barkat Mustafa, pers. comm.) enriched for stress-responsive transcripts have recently become available, allowing the construction of a boutique microarray.

The aim of the study was to utilize the pulse microarray to identify transcripts linked to biological reactions (and hence potential survival) against the major abiotic stresses of drought, cold and high-salinity in chickpea. Expression profiling of chickpea genotypes tolerant and susceptible to these abiotic stresses was performed and the transcripts differentially expressed between stressed and unstressed plants were detected. Transcripts consensually differentially expressed between stress tolerant and susceptible genotypes were identified as belonging to potentially common biological pathways.

## Results and discussion

### Experimental design and analysis

The advent of microarrays has enabled the screening of thousands of genes in parallel to assist in candidate gene identification. Ideally, one would like to scan the entire genome of a particular plant to obtain a more complete picture of transcriptional changes in response to various stresses. However, whole genome sequences are not available for most crops, leading to a dependence on collections of ESTs assembled from random cDNA libraries. For pulses, a set of chickpea unigenes, grasspea ESTs and lentil RGA sequences were recently employed for the construction of a boutique array enriched with stress-related genes [33]. Although the two main source of ESTs for this array were derived from plant tissue challenged with biotic stress (pathogen), it was clear from annotation of the ESTs that many may also be associated with abiotic stress [as seen in [34,27,29]. In fact, a considerable amount of interaction was revealed between wounding, pathogen, abiotic stress and hormonal responses in *Arabidopsis *by transcriptional profiling [35]. Many genes identified by salt-stress expression studies were reported to be common with pathogen infection [36], whilst a review on abiotic and biotic stress responses in plants [37] concluded that a significant amount of crosstalk exists in the stress signalling networks. Therefore, in the absence of a purely abiotic stress related cDNA library for chickpea, the boutique pulse array was considered an excellent tool for studying the chickpea transcriptome in response to abiotic stresses.

Expression profiling of chickpea in response to the abiotic stresses of drought, cold and high-salinity has not been previously documented. The experimental design of this study was carefully chosen to target adaptive genes, in tolerant and susceptible genotypes, by attempting to simulate natural conditions. This was achieved by cultivating plants in a glasshouse instead of growth chamber, and by applying uniform and prolonged stress before harvesting tissue samples. Moreover, it was known that chickpea is most sensitive to drought and cold stresses at flowering [38-41], thus this study examined both the leaf and flower response for drought and cold stresses. However, considering that plants usually face salinity stress from the vegetative stage (if grown on saline soils), the high-salinity stress was applied in an early growth stage. Further, the time-points chosen for tissue collection after high-salinity stress were based on the results of a pilot experiment that showed that two-week old chickpea plants could not prevent salt from reaching leaves after 48 h of stress with 150 mM NaCl (data not shown).

**Figure 1:**
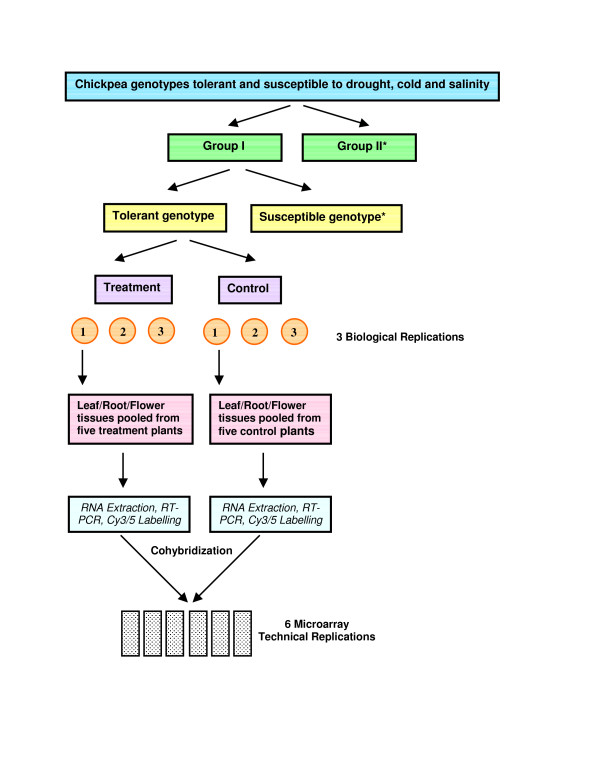
**Flow-chart showing the stress treatment procedure**. Flow-chart showing the stress treatment procedure. The high-salinity stress treatment also included two time points (24 h and 48 h) at which the tissues were harvested. *Group II was processed in the same way as Group I. Susceptible genotypes were challenged and processed in the same way as shown for tolerant genotypes.

The microarray experiments were conducted in a reference design, where tissue samples from unstressed plants acted as references against stressed plants. The tolerant and susceptible genotypes were challenged with abiotic stress in a standardized system that minimized experimental variability and ensured accurate measurements of changes in transcript abundance (Figure 1). All expression data was deposited in Minimum Information About a Microarray Experiment (MIAME) compliant format at Gene Expression Omnibus, National Center for Biotechnology Information [GEO: SuperSeries GSE7504]. The microarray observations were validated by quantitative reverse transcription-polymerase chain reaction (qRT-PCR) for several representative transcripts (Table 1). Eight genes that were commonly DE in both the tolerant/susceptible genotypes from all the stresses, time-points and tissue-types were selected for qRT-PCR validation. The comparison of expression values between the two methods revealed similar expression kinetics for all the genes tested indicating reliability of the microarray data. The expression values obtained by qRT-PCR were generally more exaggerated than the corresponding microarray values, which have also been reported in previous studies [33,42,43].

**Table 1:** Expression ratios of selected transcripts assessed by microarray and qrt-PCR. Array values indicate mean log_2 _fold change (FC) ratio relative to untreated controls and qRT-PCR values indicate log_2 _ratios of 2^(ΔC_t_control/Δ C_t _treatment). A set of DE genes that were expressed in both the tolerant/susceptible genotypes were chosen for qRT-PCR confirmation of expression.

**Treatment/Genotype/Tissue-type**	**GenBank Accession**	**Putative Function**	Group I*		Group II*	
			
			**Array**	**qPCR**	**Array**	**qPCR**
*Cold tolerant leaves*	DY475384	Similar to serine/threonine protein kinase	-2.43	-2.95	-3.27	-3.79
*Cold susceptible flowers*	DY475397	Superoxide dismutase copper chaperone precursor involved in oxidative stress	-4.16	-4.53	-1.47	-2.65
*Drought susceptible flowers*	DY475477	Asparagine synthetase (glutamine hydrolysing) (EC 6.3.5.4) – induced by the dark.	-2.66	-2.37	1.08	3.71
*Salt tolerant shoot 24 hpt*	DY475501	Chloroplast DNA for P700 chlorophyll a-apoproteins	-1.06	-2.43	-2.13	-3.56
*Salt tolerant root 24 hpt*	DY475124	Aquaporin	-1.73	-2.84	-1.00	-2.17
*Salt susceptible root 24 hpt*	DY475225	Proline oxidase enzyme involved in the conversion of proline to glutamate – induced by osmotic stress	-1.19	-1.83	-2.64	-3.12
*Salt tolerant root 48 hpt*	DY475403	Carbonic anhydrase like protein (EC 4.2.1.1) – reversible hydration of carbon dioxide	-1.47	-2.61	-2.36	-2.93
*Salt susceptible root 48 hpt*	DY475408	Xylosidase	2.48	2.73	1.09	1.67

* The tolerant/susceptible genotypes used in Group I and Group II are mentioned in Table 4.

**Figure 2:**
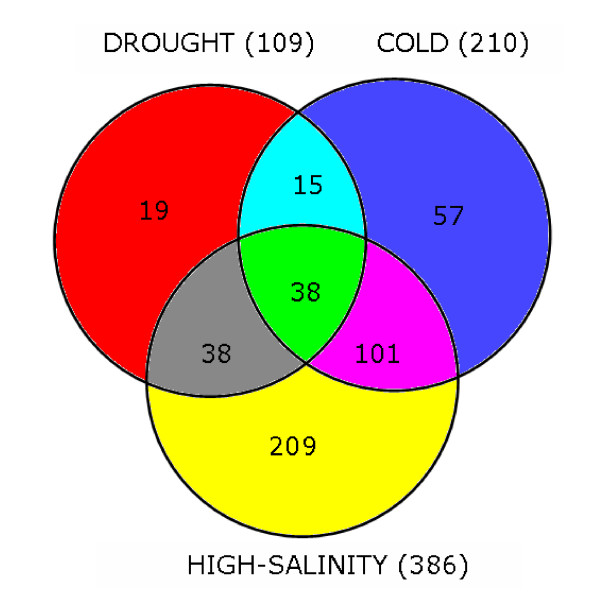
**Relationship between the number of DE transcripts in amongst the three abiotic stress treatments**. Combined relationship between the number of DE transcripts in amongst the three abiotic stress treatments for all genotypes, tissue types and time-points.

In general, the results showed that the level of several transcripts was altered by more than one of the stresses assessed (Figure 2), which may indicate gene interaction/shared pathways among the biological responses involved in these stress reactions. The number of differentially expressed (DE) transcripts affected in response to high-salinity was much higher than those affected in response to cold and drought stress in all genotypes. In *Arabidopsis*, more transcripts were revealed to be DE by drought stress (desiccation), followed by high-salinity stress (250 mM NaCl) and cold stress (4°C)[29]. However,also in *Arabidopsis*,[34] found more transcripts to be DE in response to cold stress(4°C), followed by high-salinity (100 mM NaCl) and osmotic/drought stress (200 mM Mannitol). Therefore, we propose that the number of DE transcripts in response to a particular stress depends on the method of stress induction and its severity. In this study, the lower number of DE transcripts in response to drought stress may be attributed to the nature of drought stress imposed, where pots were allowed to slowly lose water (5–10% water content/day) over a period of 8 days. This mimicked drought-stress but was relatively less severe than the cold or high-salinity stress treatments. Perhaps more DE transcripts may have been identified if the plants were held at 30% pot water content for a longer period. For all treatments, the number of undetected microarray probes (mean fluorescence intensity less than two times the mean local background intensity in all tissue-types and replications) in each chickpea genotype varied according to the source of the probes. In general, the levels of undetected features for *L. sativus *probes were higher than the *C. arietinum *probes.

None of the lentil RGA probes were detected in any treatment or genotype, possibly due to hybridization interference caused by introns present in these genomic DNA probes. Similar results were obtained [33] using the same probes for expression profiling of *Ascochyta *blight response in chickpea. The transcripts DE in response to drought, cold and high-salinity stresses included those associated with aquaporins, dehydrins, membrane-related proteins, senescence-associated proteins, superoxide dismutases, protein kinases, proline oxidase, trehalose phosphatase, phosphate-induced protein, and ubiquitins that have been previously implicated to be responsive to these stresses [44-49]. Considering the large number of DE transcripts identified for each stress, only those transcripts thought to be potentially functionally important will be focused upon ' [see Additional file 1; Additional file 2; Additional file 3]'.

### Drought stress response

Due to the poor quality and yield of RNA from root samples, only leaf and flower tissues were used for analysis of drought stress response. Six microarrays were hybridized for each of the 48 genotype × treatment/control × tissue-type × biological replication conditions, producing 288 microarray images for analysis of DE transcripts. Globally, the number of repressed transcripts was higher than those induced across all the genotypes and tissue types studied (Table 2). Tolerant genotypes shared a similar number of induced transcripts but tolerant-2 (BG 362) had twice the number of repressed transcripts than tolerant-1 (BG 1103). The susceptible-1 (Kaniva) genotype induced thrice the number of transcripts induced by susceptible-2 (Genesis 508), but both susceptible genotypes repressed the same number of transcripts. The DE transcripts in response to drought stress coded for various functional and regulatory proteins, most of which were repressed. Protein and other solute transport was repressed in susceptible-1 flowers but induced in tolerant-2 flowers. This was evidenced by the induction of a protein-transport facilitation protein (DY475074) in flowers of tolerant-2 and repression of aquaporin-like membrane channel protein (DY396334) and DNA-J like protein involved in intracellular protein transport (DY475488) in flowers of susceptible-1. Two putative auxin-repressed proteins (DY396289, DY396359) were highly repressed in flowers and leaves of tolerant-2 but were induced in flowers and leaves of susceptible-1. The plant hormone auxin regulates growth and development processes by controlling the expression of auxin-responsive genes, for example, by down-regulating auxin-repressive genes to effect growth [50]. Subsequently, the down-regulation of this gene in the tolerant genotype and up-regulation in susceptible genotype may indicate that the susceptible genotype had ceased growth due to the drought stress while the tolerant genotype was able to continue normal growth. Further, auxin-repressible genes have *cis*-elements responsive to sucrose in their promoter regions and are regulated by sucrose [50]. The sucrose-responsive transcription factor (DY475375) in this study was induced in the flowers of tolerant-2 where the auxin-repressed protein was repressed. Therefore, it may be hypothesised that sucrose plays a key role in the drought-stress response of tolerant-2.

**Table 2:** Number of > 2-fold differentially expressed transcripts for each genotype and stress. The details on transcripts > 2-fold induced or repressed are presented in Additional files 1, 2 and 3

**Condition**	**Genotype**	**Number of Transcripts**			
		
		**Induced**		**Repressed**	
***Drought***					
Tolerant-1	BG 1103	6		21	
Tolerant-2	BG 362	7		45	
Susceptible-1	Kaniva	6		20	
Susceptible-2	Genesis 508	2		21	
***Cold***					
Tolerant-1	Sonali	17		45	
Tolerant-2	ILC 01276	11		59	
Susceptible-1	Amethyst	60		43	
Susceptible-2	DOOEN	18		33	
***High-salinity***		***24 hpt***	***48 hpt***	***24 hpt***	***48 hpt***
Tolerant-1	CPI 060546	10	33	103	70
Tolerant-2	ICC 06474	23	7	85	65
Susceptible-1	CPI 60527	23	50	40	111
Susceptible-2	ICC 08161	10	7	64	62

Defence-related genes including pathogenesis-related proteins (DY396305 and DY396343), nematode-resistance protein (CV793603), *Cf-9 *gene cluster (DY396352) and disease resistance response proteins (DY396265 and DY396276) were repressed in flowers of tolerant and susceptible genotypes. Alternatively, pea disease resistance response protein (DY396390) and a multi-resistance protein ABC transporter (CV793605) were induced in flowers of both tolerant genotypes. Disease resistance proteins have been shown to be expressed in response to abiotic stresses [25], but their exact role remains unknown. Some genes involved in energy metabolism (DY396279 and DY475316) were repressed in leaves and flowers of tolerant and susceptible genotypes. The genes involved in photosynthesis were repressed in shoots following the treatment of plants with NaCl (salt stress), PEG (osmotic stress) or ABA. This response is consistent with the closure of stomata in response to high ABA or osmotic stress, inhibition of CO_2 _fixation, and a reduced need for energy capture by photosynthesis [25]. Interestingly, two cytosolic fructose 1,6-bisphosphatase transcripts involved in cellular metabolism were repressed only in flowers of both susceptible genotypes. Fructose 1,6-bisphosphatase is involved in gluconeogenesis and is subject to indirect regulation by ATP. When the concentration of ATP in the cell is low, AMP would then be high which in turn inhibits fructose 1,6-bisphosphatase and thus gluconeogenesis. Subsequently, at low ATP concentration the cell does not consume energy for synthesizing glucose. Therefore, the susceptible genotypes may be lacking ATP as a result of the drought stress. Additionally, some transcripts related to senescence, including auxin responsive protein IAA9 (DY396315), senescence-associated protein DIN 1 (DY396338), and dehydration stress-induced protein (DY396321) were repressed in leaves and flowers of the tolerant genotypes, which may also be indicative of drought tolerance.

### Cold stress response

Six microarrays were hybridized for each of the 48 genotype × treatment/control × tissue-type × biological replication conditions, producing 288 microarray images for analysis of DE transcripts. The susceptible-1 (Amethyst) genotype had the highest number of induced transcripts (Table 2), whilst tolerant-2 (ILC 01276) had the highest number of repressed transcripts. The susceptible-1 genotype was unique as it showed more induced transcripts than repressed. The number of DE transcripts in response to cold stress was approximately double than that observed for drought stress. The DE transcripts fell into various functional categories, which indicated a broad response. Important transcripts included an auxin-repressed protein (DY475078) and auxin responsive protein IAA9 (DY396315) that are involved in cell rescue and were induced in flowers and leaves of both susceptible genotypes. The induction of auxin-repressible proteins is known to be negatively correlated with shoot elongation [50], thus this observation may indicate that the growth and development of cold susceptible genotypes was repressed due to the cold-stress. Interestingly, two phosphate-induced proteins (DY475076 and DY475172) were induced in flowers/pods of tolerant-1 (Sonali). Phosphorus is important for improved flower formation and seed production, as well as earlier crop maturity [51]. This result may imply that the tolerant genotype was able to sustain flowers/pods under cold stress condition.

Interestingly, beta-glucosidase (DY475415) and beta-galactosidase (EB085056 and DY475141) transcripts were repressed in leaves of both tolerant genotypes. These enzymes are hydrolases that catalyse the reactions associated with hydrolysis of disaccharides (*e.g*. sucrose) into monosaccharides. Therefore, the tolerant genotypes appeared to be retaining disaccharides under cold stress. Further, sucrose synthase (DY475105) was induced in leaves of tolerant-1, which supports an accumulation of sucrose. Importantly, the microorganisms *Escherichia coli *and *Bacillus thuringiensis *show increased tolerance to freeze drying in the presence of disaccharides such as sucrose, and it has been proposed that they protect membranes and proteins in intact bacteria while drying [52]. Potentially, these molecules may perform a similar role in plant cells and provide protection against cold stress. Additionally, S-adenosylmethionine decarboxylase (DY475170) transcript was induced in flowers and leaves of susceptible-1 and flowers of tolerant-2 and is known to be involved in the synthesis of polyamines that act as osmolytes and accumulate under drought/osmotic stress [53].

A gluthatione S-transferase (GST) transcript (DY396404) was induced in leaves of susceptible-1 while another GST (DY475250) was repressed in leaves of tolerant-2. GST is believed to act as antioxidant enzyme to help scavenge reactive oxygen species during stress [29]. In *Arabidopsis*, two GST transcripts were induced and three were repressed in response to drought, cold and salinity [29], which indicates the variable activity of these transcripts under stress perhaps providing an array of functions in the response. Almost all the transcripts involved in energy metabolism/photosynthesis were repressed in leaves and flowers of tolerant and susceptible genotypes (*e.g*. DY475423, DY475554, DY475555, DY475487, DY475316, DY475556, DY475287, DY475434 and DY475305). This observation is not surprising since low temperature is known to cause reduced enzyme activity, which leads to impairment of photosynthesis and respiration [54,55]. Besides these, many transcripts involved in pathogen defence were induced/repressed in leaves and flowers of tolerant and susceptible genotypes (*e.g*. CV793610, DY396305, DY396390, DY475397, DY396269 and DY396359). Although defence related genes have been shown to be expressed in response to abiotic stresses [29], their actual role still remains unclear. Finally, the proteins with unknown and unclear functions that were repressed in leaves and flowers of tolerant and susceptible genotypes need further investigation to confirm their involvement and role in stress response.

### High-salinity stress response

Six microarrays were hybridized for each of the 96 genotype × treatment/control × tissue-type × time-point × biological replication conditions, producing 576 microarray images for analysis of DE transcripts. The tissues from 24 hpt and 48 hpt were analysed separately but, overall, the number of transcripts repressed were higher than those induced for all genotypes, tissue types and time points (Table 2). Tolerant-1 (CPI 060546) had the highest number of repressed transcripts at 24 hpt while susceptible-1 (CPI 60527) and tolerant-2 (ICC 06474) had the highest number of induced transcripts. At 48 hpt, susceptible-1 had highest number of induced and repressed transcripts. The number of DE transcripts detected for high-salinity stress was much higher than for both cold and drought stresses. Transcripts of interest included two poly (A) binding protein transcripts (DY396360 and DY396412) that were repressed in roots of both tolerant genotypes at 24 hpt, whilst at 48 hpt these transcripts were induced in roots and repressed in shoots of susceptible-1. Poly (A) binding proteins are a family of eukaryotic, cytoplasmic proteins thought to bind to the poly (A) tails of mRNAs and play a role in translational regulation [56]. In *Arabidopsis*, one RNA-binding protein was induced and three RNA-binding proteins repressed in response to drought, cold and high-salinity [29]. Interestingly, a splicing factor-like protein (DY396290) involved in DNA processing was repressed in roots of both tolerant genotypes at 24 hpt, and also repressed in shoots and roots of both susceptible genotypes at 24 hpt. However, at 48 hpt it was repressed only in roots of both susceptible genotypes. Subsequently, at 24 hpt, RNA production/processing may be restricted in roots/shoots of all genotypes but is repressed only in roots of susceptible genotypes at 48 hpt.

A putative heat shock protein and heat shock factor binding protein (DY396361 and DY475474) were repressed in roots and shoots of both tolerant genotypes at 24 hpt. On the contrary, the heat shock protein DNA-J homolog (DY396397) was induced in roots of susceptible-1 at 24 hpt. Further, these transcripts were repressed in roots of all tolerant and susceptible genotypes at 48 hpt. Heat shock proteins are molecular chaperones for protein molecules and play an important role in protein-protein interactions such as folding, assisting in the establishment of proper protein shape and prevention of unwanted protein aggregation. In other plants these proteins are induced by abiotic stresses like drought, cold and high-salinity [29,34]. Several of the heat shock proteins studied [29] like, HSP 90 and HSP 81-2, were repressed at 10- and 24-hpt after being induced in the first hour, thus the heat-shock proteins in this study may have been induced very early after high-salinity treatment and then repressed at the tissue sampling times. Interestingly, a proline oxidase (DY475225) transcript involved in the conversion of proline to glutamate was repressed only in roots of the susceptible genotypes at 24 hpt, and repressed in shoots and roots of susceptible-2 and in shoots of tolerant-2 at 48 hpt. Osmolytes such as proline accumulate under salt stress to prevent wilting and toxicity in the presence of high internal salt concentration and possibly aid in stress tolerance [36]. These osmolytes usually accumulate if the plants cannot maintain turgor by regulating ion exchange. Subsequently, the early repression of proline oxidase in susceptible genotypes may indicate a reaction to osmotic stress through the retention of proline, which was only observed later in one tolerant genotype.

Transcripts representing a senescence-associated protein (DY396273) and senescence associated protein DIN 1 (DY396338) were repressed in roots of tolerant-1 at 24 hpt, whilst ripening related protein (DY396347) was repressed in its shoot at this time. However, DY396338 was induced in roots of susceptible-1 at the same time-point, and DY396273 was induced in shoots of susceptible-1 at 48 hpt. These results may indicate that whilst the tolerant-1 genotype was avoiding ageing/death related genes, the susceptible-1 genotype was already undergoing cell death due to high-salinity stress at 24 hpt in roots and 48 hpt in shoots. Two cytosolic fructose 1,6-bisphosphatase transcripts (DY475548 and DY475543) were repressed only in roots of the tolerant genotypes at 24 hpt, but DY475543 was repressed in roots of susceptible genotypes at 48 hpt. As described earlier, fructose 1,6-bisphosphatase is involved in gluconeogenesis and is under indirect regulation of ATP. Thus, the roots of tolerant genotypes may be conserving energy by repressing this enzyme as early at 24 hpt, while this did not occur in the susceptible genotypes until 48 hpt, which may contribute to susceptibility.

Among the defence-related transcripts, caffeoyl-CoA O-methyltransferase 4 (DY396415), which is associated with lignification [57], was repressed in shoots and roots of both susceptible genotypes at 24 hpt, and repressed only in shoots of susceptible-1 at 48 hpt. Lignin biosynthesis is related to the reinforcement of the plant cell wall in the response to wounding or pathogen challenge. On the other hand, a putative glycine-rich cell wall protein GRP 1.8 (DY396342) was repressed only in the roots of the tolerant genotypes at 24 hpt. GRPs are also closely associated with lignification of cell walls in response to wounding or pathogen attack [58]. Thus, the repression of genes related to lignification in both susceptible and tolerant genotypes may indicate the direction of cellular resources toward other processes. Interestingly, several pathogenesis related protein 4A transcripts (DY396281, DY396372, DY396384, DY396388, CV793597) were induced in the roots of all tolerant and susceptible genotypes at 24 hpt, and again at in all genotypes except susceptible-2 at 48 hpt. Plant defence related genes have been known to be induced in response to abiotic stresses [29]. In fact, many genes identified in expression studies in response to salt stress include those in common with pathogen infection [36]. Considering that pathogenesis related protein 4A transcripts were highly induced only in response to high-salinity stress in this study, further investigation of their involvement in salt stress may be warranted.

An interesting pattern was observed amongst the transcripts related to signalling and communication. A putative histidine-containing phospho-transfer protein ATHP3 (DY396300) and a protein kinase (DY475077) were repressed only in roots of both tolerant genotypes at 24 hpt, whilst ATHP3 was repressed only in roots of tolerant-2 and protein kinase was repressed in shoots of tolerant-1 at 48 hpt. The ATHPs (or AHPs) are thought to be involved in stress sensing and relay signal transduction, where ATHP1 is thought to sense osmotic stress and transfer the signal via ATHP2/ATHP3 to the *Arabidopsis *Response Regulators (ARRs) [59]. The protein kinases are also thought to be involved in various signalling cascades related to stress responses [60]. Thus, the repression of these signalling molecules only in tolerant genotypes may have significance and needs further investigation. Putative auxin-repressed proteins (DY396269, DY396289, DY396292 and DY396359) were induced in roots of tolerant-1, tolerant-2 and susceptible-1 whilst they were repressed in shoots of tolerant-2 and susceptible-1 at 48 hpt. Auxin regulates growth and development and the induction of auxin-repressible proteins is negatively correlated with shoot elongation [50]. This observation suggests that the roots of all genotypes ceased to develop 48 hpt but the shoots were still undergoing growth, which supports the hypothesis that genes regulating cell division and elongation might be affected by salt stress [36].

Transcripts associated with transport facilitation like aquaporin (DY475124) and aquaporin-like transmembrane protein (DY396334) were repressed in roots of the tolerant genotypes at 24 hpt. Also, aquaporin 2 (integral tonoplast water channel protein; DY475512), aquaporin membrane protein (DY475174) and aquaporin-like transmembrane channel protein (DY396334) were repressed in roots of susceptible-1 at 48 hpt. At the same time only DY475174 was repressed in roots of tolerant-1. Changes in expression of aquaporins (water-channel proteins) are common to other salt stress studies and may be due to shrinkage of cells and organelles after osmotic stress [36]. Finally, many genes with unknown/unclear functions were induced/repressed in shoots and roots of all the genotypes and most of the transcripts associated with energy metabolism were repressed in all genotypes and conditions.

### Consensus stress-responsive transcripts

The main objective of this study was to identify transcripts that were consistently DE between tolerant and susceptible genotypes for each stress. Our hypothesis was that if a putative gene was consistently expressed only in tolerant or susceptible genotypes for a particular stress, it might be a candidate for tolerance/susceptibility to the stress. Of the 109 DE transcripts expressed in drought tolerant and susceptible genotypes, only two were consistently expressed (Table 3). These included a cytosolic fructose 1,6-bisphosphatase (DY475548) and a gene with unknown function, which were repressed in flowers of both susceptible genotypes. Fructose 1,6-bisphosphatase is repressed when cellular ATP levels are low to conserve energy, which may be an effect of drought susceptibility. The involvement and role of genes with unknown function have to be confirmed by additional studies.

**Table 3:** List of transcripts that were consensually differentially expressed amongst the tolerant and susceptible genotypes for each stress, tissue type and time-point

**GenBank Accession**	**Putative function**	**Log_2 _ratio**		***P *value**	
		
		Group I*	Group II*	Group I*	Group II*
*Drought susceptible flowers*					
DY475548	Cytosolic fructose 1,6-bisphosphatase	-1.7	-1.02	4.81E-43	7.33E-13
DY475051	Unknown	-1.47	-5.24	1.29E-11	7.45E-40
*Cold tolerant leaves*					
DY475555	Chlorophyll a/b binding protein	-1	-1.89	7.65E-12	1.90E-03
EB085047	18S rRNA	-1.25	-1.15	8.04E-05	1.01E-05
DY396262	Probable Ca-binding mitochondrial carrier	-1.1	-1.6	2.82E-11	3.97E-03
DY475384	Serine/threonine protein kinase	-2.43	-3.27	1.19E-18	1.33E-08
DY475141	Beta-galactosidase	-2	-1.63	1.35E-05	2.98E-04
DY396371	Polyubiquitin	-1.05	-1.62	2.62E-08	7.42E-04
DY475282	Trehalose-phosphatase	-1.27	-1.82	2.94E-04	3.49E-05
DY396343	Pathogenesis-related protein	-1.46	-1.69	1.05E-11	3.27E-06
DY396307	Serine/threonine protein kinase	-1.12	-1.14	1.93E-18	4.64E-03
DY475323	Unclear	-1.42	-2.05	2.53E-05	2.84E-28
DY475203	Unknown	-1.51	-1.6	4.58E-04	3.91E-05
*Cold susceptible leaves*					
DY475523	Sorting nexin protein	-2.1	-1.12	6.18E-05	8.43E-05
DY475329	Unclear	-1.7	-1.07	1.92E-05	8.39E-03
DY475431	Unknown	-1.54	-1.63	8.43E-03	3.79E-06
*Cold susceptible flowers*					
DY475397	Superoxide dismutase copper chaperone precursor	-4.16	-1.47	9.50E-07	7.69E-09
*High-salinity tolerant shoot 24 hpt*					
DY475501	P700 chlorophyll a-apoprotein	-1.06	-2.13	1.06E-03	1.13E-06
DY475287	NADH-plastoquinone oxidoreductase subunit I	-3.55	-2.41	3.75E-04	1.53E-04
DY475215	Unknown	-2.14	-1.52	3.97E-07	2.61E-06
*High-salinity tolerant root 24 hpt*					
EB085052	Unknown	-1.46	-1.64	3.69E-07	1.78E-08
DY396290	Splicing factor-like protein	-1.7	-1.31	1.75E-03	1.22E-02
DY396300	ATHP3 (histidine-containing phosphotransfer protein)	-1.3	-1.21	3.40E-06	1.24E-10
DY396342	Glycine-rich cell wall protein GRP 1.8	-1.43	-2.77	3.51E-03	4.01E-04
DY475077	Protein kinase	-1.37	-2.91	2.31E-07	2.13E-06
DY475548	Cytosolic fructose 1,6-bisphosphatase	-2.36	-1.22	2.10E-03	5.54E-04
DY475124	Aquaporin	-1.73	-1	9.28E-13	1.57E-13
DY475256	Unknown	-1.07	-1.15	1.77E-05	1.31E-02
DY475275	Unknown	-1.93	-1.27	2.68E-10	3.50E-05
DY475293	Unknown	1.23	1.4	4.70E-06	8.35E-11
DY475347	Unknown	-5.82	-2.72	2.49E-03	1.58E-19
DY475416	Unknown	-1.94	-1.67	9.14E-03	3.24E-04
*High-salinity susceptible root 24 hpt*					
DY475225	Proline oxidase	-1.19	-2.64	5.03E-04	1.08E-08
DY475186	Unclear	-2.3	-1.56	4.35E-09	1.36E-08
*High-salinity tolerant shoot 48 hpt*					
DY396301	Pathogenesis-related protein	-3.26	-1.73	2.87E-08	3.30E-07
*High-salinity susceptible shoot 48 hpt*					
DY475205	Unclear	2.49	1.45	2.18E-16	1.63E-04
DY475048	Unknown	-2.7	-1.76	6.97E-03	3.47E-03
*High-salinity tolerant root 48 hpt*					
DY396262	Probable Ca-binding mitochondrial carrier	-1.18	-1.26	9.17E-15	7.88E-03
DY475403	Carbonic anhydrase	-1.47	-2.36	5.46E-07	2.28E-09
DY475242	Thiazole biosynthetic enzyme	-1.77	-2.48	4.38E-06	9.27E-06
DY396281	Pathogenesis-related protein 4A	3.35	2.6	2.81E-17	3.49E-14
DY475416	Unknown	-3.71	-2.25	4.30E-06	1.80E-08
DY475521	Unknown	1.38	1.01	3.68E-09	6.70E-07
*High-salinity susceptible root 48 hpt*					
DY396290	Splicing factor-like protein	-2.63	-1.65	4.68E-08	1.97E-04
DY475408	Xylosidase	2.48	1.09	9.41E-04	4.53E-03
DY475217	Unclear	-2.03	-1.02	1.39E-04	7.44E-03
DY475390	Unknown	2.19	1.05	2.65E-04	1.56E-03

* The tolerant/susceptible genotypes used in Group I and Group II are mentioned in Table 4.

Fifteen out of the 210 DE transcripts found in cold tolerant and susceptible genotypes were consistently expressed (Table 3), all of which were repressed. Most of the putative genes were identified in leaves of the tolerant genotypes, and included a beta-galactosidase (DY475141) transcript that was described earlier as possibly indicative of disaccharide (*e.g*. sucrose) retention with the effect of protecting cell membranes during cold stress. Several protein synthesis/modification and energy/metabolism transcripts were also repressed (*e.g*. DY475282, DY396371 and DY475555), which was likely due to the impairment of photosynthesis and respiration at low temperature [54,55]. Other consistently repressed transcripts in tolerant genotypes included putative signalling (DY396262, DY475384 and DY396307) and defence-related proteins (CV793589 and DY396343), which may be involved in the repression of cell death mechanisms. In susceptible genotypes, a putative superoxide dismutase precursor protein (DY475397) and sorting nexin protein (DY475523) were the only known transcripts to be consistently repressed. Superoxide dismutase is involved in the programmed cell death pathway where its repression allows the accumulation of reactive oxygen species that signal and contribute to cell death [61]. Subsequently, this result suggests that cold stress in susceptible genotypes may lead to the promotion of cell death pathways.

Of the transcripts consistently expressed in tolerant genotypes in response to high-salinity stress, the annotation of transcripts at 24 hpt suggest a reduction in energy production in shoots and roots by repression of putative genes including P700 chlorophyll a-apoprotein (DY475501) and NADH-plastoquinone oxidoreductase subunit I (DY475287), cytosolic fructose 1,6-bisphosphatase (DY475548) and splicing factor-like protein (DY396290). These observations may indicate that the available cellular resources have instead been deployed to maintain the ionic balance needed to tolerate the high-salinity conditions. The ATHP3 (DY396300) and protein kinase (DY475077) are potentially involved in signalling cascades responsible for sensing and relaying osmotic stress signals, but their consistent repression in tolerant genotypes suggest that they may negatively regulate the genes responsible for signalling high-salinity tolerance. Additionally a glycine rich protein (DY396342) that is associated with lignification of cell walls in response to wounding and pathogen attack was repressed in tolerant genotypes, which indicates that a reduction of lignin deposition may be an effect of high-salinity tolerance. In tolerant genotypes at 48 hpt, two energy and metabolic-related transcripts were consistently repressed, including a carbonic anhydrase (DY475403) and putative thiazole biosynthetic enzyme (DY475242) in roots, suggesting again the sacrifice of general cellular functions for maintenance of ionic balance. In shoots, only a pathogenesis-related protein (DY396301) was consistently repressed at 48 hpt, but a similar transcript (DY396281) was induced in roots of tolerant genotypes at the same time. As described earlier, PR proteins have been shown to be expressed under abiotic stresses but their role is not very well understood. Subsequently, these putative defence-related proteins may have a tissue-specific role in conferring high-salinity tolerance in chickpea.

Only two transcripts were DE in susceptible genotypes at 24 hpt, all occurring in root tissue. One of these was gene with unclear function and the other a proline oxidase transcript (DY475225) that may indicate the retention of proline, which is an osmolyte known to accumulate under osmotic stresses and plays a role in stabilizing structure of plant proteins [36]. At 48 hpt, a putative splicing factor-like protein (DY396290) was consistently repressed in roots of susceptible genotypes, suggesting the potential stress-related repression of RNA production/processing. Interestingly, a putative xylosidase (DY475408) was induced in susceptible roots at 48 hpt. Xylosidase exhibits hydrolytic activity towards polysaccharides and is responsible for structural changes by degradation of polysaccharides to allow the modification of the cell wall [62]. Thus, this result shows that susceptible genotypes may undergo cell wall degradation as a result of high-salinity stress. Finally, several unknown/unclear transcripts were DE in both tolerant and susceptible genotypes. Of interest were two unknowns (DY475293 and DY475521) that were consistently induced in tolerant genotypes and may be important for high-salinity tolerance.

## Conclusion

This study revealed that 476 transcripts were DE in all stresses, genotypes, tissue-types, and time points tested. The large number of transcriptome changes observed highlights the difficulty of understanding the global context of stress responses. In *Arabidopsis*, approximately 30% of the genome was potentially regulated by salt, cold and osmotic stress [34]. In our study it was also observed that the number of transcripts expressed depended on the type and severity of stress, where more transcripts were expressed under high-salinity followed by cold and drought stress. The DE transcripts between the stressed and unstressed plants were classified in relation to functional categories and, overall, more genes were found to be repressed than induced. The genes that were consistently DE in groups of tolerant and susceptible genotypes for each stress were compiled and interesting observations were made when the DE genes were analysed with respect to their biological role in plants.

The main objective of this study was to identify a suite of putative genes that were consistently expressed in tolerant or susceptible genotypes for each stress condition. To our knowledge, this is the first intensive cDNA microarray study for abiotic stress responses in chickpea. Several candidate genes for tolerance/susceptibility were identified from Table 3, but it is crucial to emphasize that changes in mRNA accumulation may not necessarily correlate with protein/enzyme activity levels [63]. Moreover, when applying stress treatments, the response in the plant may be variable due to the nature of treatment, variation in response by plants, or natural variation between plants [28]. Therefore, it may possibly be ideal to compare expression profiles of recombinant inbred lines (RILs) or near isogenic lines (NILs) that are tolerant and susceptible to these abiotic stresses to reduce background genetic variation amongst the plants. The expression profiles provide starting points for in-depth studies on candidate genes to help prioritize the intensive task of using reverse genetics to assign gene functions [34]. The results of this study should therefore be carefully extrapolated until further in-depth studies have been carried out. Nevertheless, the annotation of transcripts with significant fold change and detection of consistently DE transcripts between tolerant and susceptible genotypes strongly suggests that these putative genes have a role in abiotic stress responses. Subsequently, the experimental set-up and downstream analysis methods applied in this study are appropriate for identification of putative stress induced transcripts in chickpea. The identification of novel genes, determination of their expression patterns in response to different stress conditions, and an improved understanding of their functions in stress adaptation will provide basic knowledge to design effective engineering strategies for enhancement of stress tolerances.

## Methods

### Plant materials and experimental design

Chickpea genotypes tolerant and susceptible to the abiotic stresses of drought, cold and high-salinity were selected (Dr. B. Redden, 2005, pers. comm.; Dr. H. Clarke, 2005, pers. comm.; Moses Maliro, 2005, pers. comm.) and obtained from the Australian Temperate Field Crops Collection, Horsham, Australia (Table 4). Two groups of a tolerant and susceptible genotype were screened to generate an expression profile in response to each abiotic stress. The stress treatments were performed on the tolerant and susceptible genotypes in three biological replications. The experiments were conducted in reference design where respective tissues from unstressed plants served as control. The genes expressed by the two tolerant and susceptible genotypes (for each stress/tissue-type/time-point) were compared in a two-way comparison to reveal genes that were consistently expressed only in the tolerant/susceptible accessions.

**Table 4:** List of abiotic stress tolerant and susceptible genotypes used in two groups of stress experiments. Genotypes are listed by common name and Australian Temperate Crop (ATC) identification number

**Characteristic**	**Group I**	**Group II**
Drought tolerant	BG 1103 (ATC 48111)	BG 362 (ATC 48104)
Drought susceptible	Kaniva (ATC 40030)	Genesis 508 (ATC 45226)
Cold tolerant	Sonali (ATC 48113)	ILC 01276 (ATC 40021)
Cold susceptible	Amethyst (ATC 42331)	DOOEN (ATC 40874)
Salt tolerant	CPI 060546 (ATC 40586)	ICC 06474 (ATC 40171)
Salt susceptible	CPI 60527 (ATC 40033)	ICC 08161 (ATC 40707)

* The tolerant/susceptible genotypes used in Group I and Group II are mentioned in Table 4.

### Drought stress treatments

The drought tolerant and susceptible genotypes (five treatment and five control plants per genotype) were cultivated (one plant per 15 cm pot) in sterile potting mix in a glasshouse at 15–25°C. All plants were watered to keep the soil moist but excess watering was avoided. The plants were fertilized twice with urea during establishment and once with Nitrosol^® ^(Amgrow, Australia) 45 days after sowing. The drought stress was imposed two weeks after flowering as follows: All plants were saturated with water late in the evening, and the following morning pots were bagged so that no water was allowed to evaporate. A 1.0 ml pipette tip was cut slightly at the tip and inserted in the pot to allow addition of water. The initial pot weight was recorded. The water content in each pot was estimated to be 30% of the initial pot weight (based on wet weight and dry weight). From the subsequent day onwards, the control pots were maintained at 80% water content and the treatment pots were allowed to lose 5–10% of their water content per day and any extra water lost (> 10%) was replenished. The leaf, root and flower/bud tissues were collected separately when the treatment pots reached 30% water content, indicative of a drought or high water deficit condition [64] (Dr. V. Vincent, 2005, pers. comm.; Dr. D. Hoisington, 2005, pers comm.). The tissues from the control plants were also collected at this time. All tissues were snap frozen in liquid nitrogen and preserved at -80°C until RNA extraction.

### Cold stress treatments

The cold tolerant and susceptible genotypes (five treatment and five control plants per genotype) were cultivated as described for the drought stress treatment until the cold stress treatment commenced two weeks after flowering. To simulate cold stress, treatment plants were exposed to a 12 h day/12 h night temperature cycle of 15–25°C and 5°C [38,65] (Dr. H. Clarke, 2005, pers comm.). The control plants were maintained in glasshouse conditions (15–25°C). The leaf and flowers/buds/immature pod tissues from treatment plants were collected after the seventh night at 5°C. The tissues from the control plants were also collected at this time and all tissues were snap frozen in liquid nitrogen and preserved at -80°C until RNA extraction.

### High-salinity stress treatments

The high-salinity tolerant and susceptible genotypes were cultivated in a hydroponic system using 50 L plastic crates. Two crates were set-up, one each for treatment and control. Forty holes (8 × 5 grid) of 5 cm diameter were drilled in the crates' lid and rock wool plugs were placed in them. Ten pre-germinated seeds per tolerant and susceptible genotype were transplanted in alternate plugs within each crate. The seedlings were watered normally from above for four days. The following day, the crates were filled with 0.5 × modified Hoagland's nutrient medium (pH 6.5) [66]. The medium was aerated using two aquarium pumps per crate. The nutrient medium was subsequently replaced with 1.0 × Hoagland's solution (pH 6.5) after a further seven days. At day 18, the nutrient medium for the treatment plants was replaced with 1.0 × modified Hoagland's with 150 mM Sodium Chloride (NaCl) (pH 6.5), which represented a salinity concentration known to be toxic to chickpea [6] (unpublished data). The control plants continued to grow in replaced 1.0 × modified Hoagland's solution (pH 6.5). Leaf/shoot and root tissues were collected from five treatment plants at 24 and 48 h post-treatment (hpt). The tissues from control plants were also collected at these times. The tissues were snap frozen in liquid nitrogen and preserved at -80°C until RNA extraction.

### Microarray construction, target preparation and hybridization

Microarrays were constructed in accordance with MIAME guidelines [67], following the method of [33]. The 768-feature microarrays consisted of 559 chickpea cDNAs, 156 grasspea cDNAs, 41 lentil resistance gene analogs (RGAs) and 12 controls. A complete description of the microarray features can be found. ' [see Additional file 4]'. Printed microarrays were pre-hybridized by blocking in 5 × SSC, 0.1% SDS, 25% formamide, 1% BSA for 45 min at 42°C, rinsed in distilled water and dried. Total RNA was extracted from separately pooled tissue samples for each treatment, followed by labeling and hybridization to microarray slides according to [33]. Dye swap was performed in one of the three biological replicates.

### Scanning and data analysis

Slides were scanned and images captured as described [33]. Data transformations consisted of a local background correction, omitting flagged spots, normalization by applying the LOWESS algorithm [68], creating a Cy5/Cy3 mean signal ratio, log_2 _conversion, and combining replicates. To identify differentially expressed (DE) genes, expression ratio results were filtered to eliminate genes whose 95% confidence interval for mean fold change (FC) did not extend to 2-fold up or down. These cut-offs translated into induced transcripts having a log_2 _ratio ≥ 1.0 and repressed transcripts a ratio of ≤ -1.0. This was followed by a Students *t *test and False Discovery Rate (FDR) multiple testing correction to retain only genes in which expression changes *v*. unstressed control were significant at P < 0.05. High data quality and reproducibility was achieved using five experimental replicates (five stressed and five unstressed plants), three biological replicates and six technical replicates for all microarray spots. Data quality was also improved by the inclusion of negative controls and a dye-swap for one biological replicate.

### Quantitative Reverse -Transcription PCR (qRT-PCR)

The microarray expression results were validated by performing qRT-PCR on a set of selected DE transcripts. This set was chosen to represent different stresses, genotypes, tissue-types, time points and expression values (up/down-regulation). The PCR primers were designed using Primer3 [69] and had a GC content of 40–60%, a Tm > 50°C, a primer length 20–25 nucleotides, and an expected amplicon size was 100–250 bp. The comparative Ct method of quantitation was used with the Actin gene (DY475300) as a reference. The relative fold-change for each of the selected genes was detected from both the tolerant/susceptible genotypes. For each genotype, 5 μg total RNA from one of the biological replicates was converted into cDNA using oligodT 15-mer (Roche Diagnostics, Mannheim, Germany) and Superscript II reverse transcriptase (Invitrogen Life Technologies, Carlsbad, CA). This cDNA was purified using the Qiaquick PCR purification kit (Qiagen, Valencia, CA) and diluted to 250 μL in sterile water. Validation experiments were performed on 5 to 6 log dilutions of each of the target genes together with the Actin reference to determine if the amplification efficiencies were equal. Triplicate qRT-PCR reactions were performed using iQ™SYBR^® ^Green Supermix (Bio-Rad, Hercules, CA),0.4 μM of forward and reverse primers and the required amounts of cDNA template. The PCRs were performed in a Bio-Rad MyiQ™ thermocycler (Bio-Rad, Hercules, CA). The temperature regime used was 95°C for 10 m followed by 40 cycles of 30 s at 95°C, 45 s at 55°C and 45 s at 72°C. Melting curve analysis by applying increasing temperature from 45°C to 95°C (0.5°C/10 s) and gel and gel electrophoresis of final product confirmed single amplicons. Negative control reactions using untranscribed RNA were also run to confirm absence of genomic DNA. The relative fold change for a particular target was determined by comparing the Ct values for the treatment with that of the control. The Ct values were normalized using the Ct reference (actin) prior to comparison.

## Authors' contributions

NM carried out most of the work described here including conception and design of experiments, acquisition of data, analysis and interpretation of data and drafting the manuscript. RF and EP contributed towards conception and design of experiments, supervision of the work and critical review of the manuscript. TC contributed towards design of experiments, analysis and interpretation of data and review of manuscript. All authors read and approved the final manuscript.

## Supplementary Material

Additional file 1 Genes > 2-fold differentially expressed between drought stressed and unstressed plants (sorted with respect to their putative function). The genes > 2-fold differentially expressed between drought stressed and unstressed plants. The table has been sorted with respect to putative function of genes and details of genotype/tissue-type in which the genes were expressed are given. Click here for file

Additional file 2 Genes > 2-fold differentially expressed between cold stressed and unstressed plants (sorted with respect to their putative function). The genes > 2-fold differentially expressed between cold stressed and unstressed plants. The table has been sorted with respect to putative function of genes and details of genotype/tissue-type in which the genes were expressed are given. Click here for file

Additional file 3 Genes > 2-fold differentially expressed between high-salinity stressed and unstressed plants (sorted with respect to their putative function). The genes > 2-fold differentially expressed between high-salinity stressed and unstressed plants. The table has been sorted with respect to putative function of genes and details of genotype/tissue-type in which the genes were expressed are given. Click here for file

Additional file 4 Characteristics of the 768 microarray features. The details of the 768 features printed on the spotted cDNA array. It includes the Spot position, GenBank^® ^Accession, Gene Name, Source, Biosequence type and its usage (reporter/control). Click here for file
