# Supplementary material for: Transcriptional profiling of chickpea genes differentially regulated in response to high-salinity, cold and drought
Source: BMC Genomics. 2007 Sep 2;8:303. doi: 10.1186/1471-2164-8-303 (PMC2025592; doi:10.1186/1471-2164-8-303)
Supplement: Additional file 4 — Characteristics of the 768 microarray features. The details of the 768 features printed on the spotted cDNA array. It includes the Spot position, GenBank® Accession, Gene Name, Source, Biosequence type and its usage (reporter/control). [file 1471-2164-8-303-S4.pdf]

Additional file 4. Characteristics of the 768 microarray features.

Meta Row	Meta Column	Row	Column	GenBank Accession	Gene Name	Source	Biosequence Type	Reporter Usage	Control Type
1	1	1	1	DY396334	Aquaporin-like transmembrane channel protein	*Lathyrus sativus*	cDNA clone	Experimental	NA
1	1	1	2	DY396423	Gibberellin-regulated protein 3 precursor	*Lathyrus sativus*	cDNA clone	Experimental	NA
1	1	1	3	NA	NBS-LRR putative RGA Aj516088	*Lens culinaris* (ILL7537)	Genomic PCR product	Experimental	NA
1	1	1	4	NA	Lipoxygenase	*Lens culinaris* (ILL7537)	Genomic PCR product	Experimental	NA
1	1	1	5	NA	NBS-LRR putative RGA LR1	*Lens culinaris* (ILL7537)	Genomic PCR product	Experimental	NA
1	1	1	6	NA	NBS-LRR putative RGA Aj516061	*Lens culinaris* (ILL6002)	Genomic PCR product	Experimental	NA
1	1	1	7	NA	NBS-LRR putative RGA Aj516063	*Lens culinaris* (ILL6002)	Genomic PCR product	Experimental	NA
1	1	1	8	NA	NBS-LRR putative RGA Aj516065	*Lens culinaris* (ILL6002)	Genomic PCR product	Experimental	NA
1	1	1	9	NA	NBS-LRR putative RGA Aj516070	*Lens culinaris* (ILL6002)	Genomic PCR product	Experimental	NA
1	1	1	10	NA	NBS-LRR putative RGA Aj516073	*Lens culinaris* (ILL6002)	Genomic PCR product	Experimental	NA
1	1	2	1	DY396360	Poly(A)-binding protein	*Lathyrus sativus*	cDNA clone	Experimental	NA
1	1	2	2	DY396394	Transcription initation factor TFIID 85 KDA subunit	*Lathyrus sativus*	cDNA clone	Experimental	NA
1	1	2	3	DY396387	Similarity to RNA-binding protein	*Lathyrus sativus*	cDNA clone	Experimental	NA
1	1	2	4	DY396378	Polyubiquitin	*Lathyrus sativus*	cDNA clone	Experimental	NA
1	1	2	5	DY396376	Polyubiquitin	*Lathyrus sativus*	cDNA clone	Experimental	NA
1	1	2	6	DY396371	Polyubiquitin	*Lathyrus sativus*	cDNA clone	Experimental	NA
1	1	2	7	DY396414	Splicing factor RSZ33	*Lathyrus sativus*	cDNA clone	Experimental	NA
1	1	2	8	DY396410	Polyubiquitin	*Lathyrus sativus*	cDNA clone	Experimental	NA
1	1	2	9	DY396293	Thioredoxin	*Lathyrus sativus*	cDNA clone	Experimental	NA
1	1	2	10	DY396290	Splicing factor-like protein	*Lathyrus sativus*	cDNA clone	Experimental	NA
1	1	2	11	DY396282	18.2 KDA class I heat shock protein	*Lathyrus sativus*	cDNA clone	Experimental	NA
1	1	2	12	DY396279	NADH dehydrogenase	*Lathyrus sativus*	cDNA clone	Experimental	NA
1	1	2	13	DY396386	Amine oxidase	*Lathyrus sativus*	cDNA clone	Experimental	NA
1	1	2	14	DY396338	Senescence-associated protein DIN1	*Lathyrus sativus*	cDNA clone	Experimental	NA
1	1	3	1	DY396374	Subtilisin inhibitors I and II (ASI-I and ASI-II)	*Lathyrus sativus*	cDNA clone	Experimental	NA
1	1	3	2	DY396379	Putative auxin-repressed protein	*Lathyrus sativus*	cDNA clone	Experimental	NA
1	1	3	3	DY396382	Protein kinase-like protein	*Lathyrus sativus*	cDNA clone	Experimental	NA
1	1	3	4	DY396405	PR1A precursor	*Lathyrus sativus*	cDNA clone	Experimental	NA
1	1	3	5	DY396389	Polygalacturonase inhibitor protein	*Lathyrus sativus*	cDNA clone	Experimental	NA
1	1	3	6	DY396392	Multi resistance protein (F20D22.11 protein)	*Lathyrus sativus*	cDNA clone	Experimental	NA
1	1	3	7	DY396288	Hypothetical proline-rich protein	*Lathyrus sativus*	cDNA clone	Experimental	NA
1	1	3	8	DY396302	Polyubiquitin	*Lathyrus sativus*	cDNA clone	Experimental	NA
1	1	3	9	DY396432	Transcription initiation factor IIF, beta subunit	*Lathyrus sativus*	cDNA clone	Experimental	NA

1	1	3	10	DY396286	Ubiquitin	*Lathyrus sativus*	cDNA clone	Experimental	NA
1	1	3	11	DY396274	Ubiquitin-specific protease 6	*Lathyrus sativus*	cDNA clone	Experimental	NA
1	1	3	12	DY396322	Metallothionein-like protein 1	*Lathyrus sativus*	cDNA clone	Experimental	NA
1	1	3	13	DY396320	salt-inducible protein-like	*Lathyrus sativus*	cDNA clone	Experimental	NA
1	1	3	14	DY396318	Transcription initiation factor IIF beta subunit	*Lathyrus sativus*	cDNA clone	Experimental	NA
1	1	4	1	NA	Unknown	*Cicer arietinum*	cDNA clone	Experimental	NA
1	1	4	2	EB085055	26S rRNA	*Cicer arietinum*	cDNA clone	Experimental	NA
1	1	4	3	NA	Unknown	*Cicer arietinum*	cDNA clone	Experimental	NA
1	1	4	4	EB085058	Unclear	*Cicer arietinum*	cDNA clone	Experimental	NA
1	1	4	5	DY396283	Protein kinase C inhibitor-like protein	*Lathyrus sativus*	cDNA clone	Experimental	NA
1	1	4	6	DY396289	Putative auxin-repressed protein	*Lathyrus sativus*	cDNA clone	Experimental	NA
1	1	4	7	DY396292	Putative auxin-repressed protein	*Lathyrus sativus*	cDNA clone	Experimental	NA
1	1	4	8	DY396296	Disease resistance response protein 39 precursor	*Lathyrus sativus*	cDNA clone	Experimental	NA
1	1	4	9	DY396299	Beta-glucan binding protein	*Lathyrus sativus*	cDNA clone	Experimental	NA
1	1	4	10	DY396301	Pathogenesis-related protein	*Lathyrus sativus*	cDNA clone	Experimental	NA
1	1	4	11	DY396305	Pathogenesis-related protein	*Lathyrus sativus*	cDNA clone	Experimental	NA
1	1	4	12	DY396311	Disease resistance response protein 230 precursor	*Lathyrus sativus*	cDNA clone	Experimental	NA
1	1	4	13	DY396365	Serine acetyl transferase	*Lathyrus sativus*	cDNA clone	Experimental	NA
1	1	4	14	DY396369	Putative WD-repeat protein	*Lathyrus sativus*	cDNA clone	Experimental	NA
1	1	5	1	EB085019	Chloroplast DNA	*Cicer arietinum*	cDNA clone	Experimental	NA
1	1	5	2	EB085021	Unknown	*Cicer arietinum*	cDNA clone	Experimental	NA
1	1	5	3	EB085038	Chloroplast DNA	*Cicer arietinum*	cDNA clone	Experimental	NA
1	1	5	4	EB085039	Unknown	*Cicer arietinum*	cDNA clone	Experimental	NA
1	1	5	5	DY475538	Unknown	*Cicer arietinum*	cDNA clone	Experimental	NA
1	1	5	6	NA	Unknown	*Cicer arietinum*	cDNA clone	Experimental	NA
1	1	5	7	NA	Unknown	*Cicer arietinum*	cDNA clone	Experimental	NA
1	1	5	8	EB085043	Translation initiation factor SUI1	*Cicer arietinum*	cDNA clone	Experimental	NA
1	1	5	9	NA	Unknown	*Cicer arietinum*	cDNA clone	Experimental	NA
1	1	5	10	EB085045	Unclear	*Cicer arietinum*	cDNA clone	Experimental	NA
1	1	5	11	EB085051	Unknown	*Cicer arietinum*	cDNA clone	Experimental	NA
1	1	5	12	EB085066	4.5S, 5S, 16S and 23S rRNA	*Cicer arietinum*	cDNA clone	Experimental	NA
1	1	5	13	EB085053	Unknown	*Cicer arietinum*	cDNA clone	Experimental	NA
1	1	5	14	NA	Unknown	*Cicer arietinum*	cDNA clone	Experimental	NA
1	1	6	1	NA	Unknown	*Cicer arietinum*	cDNA clone	Experimental	NA
1	1	6	2	NA	Unknown	*Cicer arietinum*	cDNA clone	Experimental	NA
1	1	6	3	NA	Unknown	*Cicer arietinum*	cDNA clone	Experimental	NA

1	1	6	4	NA	Unknown	*Cicer arietinum*	cDNA clone	Experimental	NA
1	1	6	5	EB085060	Unknown	*Cicer arietinum*	cDNA clone	Experimental	NA
1	1	6	6	NA	Unknown	*Cicer arietinum*	cDNA clone	Experimental	NA
1	1	6	7	EB085027	5.8S, 18S and 25S rRNA	*Cicer arietinum*	cDNA clone	Experimental	NA
1	1	6	8	NA	Unknown	*Cicer arietinum*	cDNA clone	Experimental	NA
1	1	6	9	NA	Unknown	*Cicer arietinum*	cDNA clone	Experimental	NA
1	1	6	10	EB085065	18S rRNA	*Cicer arietinum*	cDNA clone	Experimental	NA
1	1	6	11	DY475554	Chlorophyll a/b binding protein	*Cicer arietinum*	cDNA clone	Experimental	NA
1	1	6	12	NA	Unknown	*Cicer arietinum*	cDNA clone	Experimental	NA
1	1	6	13	DY475536	Unknown	*Cicer arietinum*	cDNA clone	Experimental	NA
1	1	6	14	DY475532	Unknown	*Cicer arietinum*	cDNA clone	Experimental	NA
1	1	7	1	DY475350	Unknown	*Cicer arietinum*	cDNA clone	Experimental	NA
1	1	7	2	DY475353	Unknown	*Cicer arietinum*	cDNA clone	Experimental	NA
1	1	7	3	DY475360	Unknown	*Cicer arietinum*	cDNA clone	Experimental	NA
1	1	7	4	DY475363	Unknown	*Cicer arietinum*	cDNA clone	Experimental	NA
1	1	7	5	DY475365	Unknown	*Cicer arietinum*	cDNA clone	Experimental	NA
1	1	7	6	DY475369	Unknown	*Cicer arietinum*	cDNA clone	Experimental	NA
1	1	7	7	DY475436	Unknown	*Cicer arietinum*	cDNA clone	Experimental	NA
1	1	7	8	DY475439	Unknown	*Cicer arietinum*	cDNA clone	Experimental	NA
1	1	7	9	DY475446	Unknown	*Cicer arietinum*	cDNA clone	Experimental	NA
1	1	7	10	DY475459	Unknown	*Cicer arietinum*	cDNA clone	Experimental	NA
1	1	7	11	DY475462	Unknown	*Cicer arietinum*	cDNA clone	Experimental	NA
1	1	7	12	DY475472	Unknown	*Cicer arietinum*	cDNA clone	Experimental	NA
1	1	7	13	DY475481	Unknown	*Cicer arietinum*	cDNA clone	Experimental	NA
1	1	7	14	DY475483	Unknown	*Cicer arietinum*	cDNA clone	Experimental	NA
1	1	8	1	DY475171	Unknown	*Cicer arietinum*	cDNA clone	Experimental	NA
1	1	8	2	DY475178	Unknown	*Cicer arietinum*	cDNA clone	Experimental	NA
1	1	8	3	DY475187	Unknown	*Cicer arietinum*	cDNA clone	Experimental	NA
1	1	8	4	DY475191	Unknown	*Cicer arietinum*	cDNA clone	Experimental	NA
1	1	8	5	DY475260	Unknown	*Cicer arietinum*	cDNA clone	Experimental	NA
1	1	8	6	DY475268	Unknown	*Cicer arietinum*	cDNA clone	Experimental	NA
1	1	8	7	DY475275	Unknown	*Cicer arietinum*	cDNA clone	Experimental	NA
1	1	8	8	DY475279	Unknown	*Cicer arietinum*	cDNA clone	Experimental	NA
1	1	8	9	DY475281	Unknown	*Cicer arietinum*	cDNA clone	Experimental	NA
1	1	8	10	DY475288	Unknown	*Cicer arietinum*	cDNA clone	Experimental	NA
1	1	8	11	DY475291	Unknown	*Cicer arietinum*	cDNA clone	Experimental	NA

1	1	8	12	DY475295	Unknown	*Cicer arietinum*	cDNA clone	Experimental	NA
1	1	8	13	DY475342	Unknown	*Cicer arietinum*	cDNA clone	Experimental	NA
1	1	8	14	DY475347	Unknown	*Cicer arietinum*	cDNA clone	Experimental	NA
1	1	9	1	DY475323	Unclear	*Cicer arietinum*	cDNA clone	Experimental	NA
1	1	9	2	DY475333	Unclear	*Cicer arietinum*	cDNA clone	Experimental	NA
1	1	9	3	DY475552	Unclear	*Cicer arietinum*	cDNA clone	Experimental	NA
1	1	9	4	DY475522	Unclear	*Cicer arietinum*	cDNA clone	Experimental	NA
1	1	9	5	DY475528	Unclear	*Cicer arietinum*	cDNA clone	Experimental	NA
1	1	9	6	DY475054	Unknown	*Cicer arietinum*	cDNA clone	Experimental	NA
1	1	9	7	DY475056	Unknown	*Cicer arietinum*	cDNA clone	Experimental	NA
1	1	9	8	DY475062	Unknown	*Cicer arietinum*	cDNA clone	Experimental	NA
1	1	9	9	DY475067	Unknown	*Cicer arietinum*	cDNA clone	Experimental	NA
1	1	9	10	DY475079	Unknown	*Cicer arietinum*	cDNA clone	Experimental	NA
1	1	9	11	DY475157	Unknown	*Cicer arietinum*	cDNA clone	Experimental	NA
1	1	9	12	DY475159	Unknown	*Cicer arietinum*	cDNA clone	Experimental	NA
1	1	9	13	DY475165	Unknown	*Cicer arietinum*	cDNA clone	Experimental	NA
1	1	9	14	DY475167	Unknown	*Cicer arietinum*	cDNA clone	Experimental	NA
1	1	10	1	DY475209	Lipid transfer protein	*Cicer arietinum*	cDNA clone	Experimental	NA
1	1	10	2	DY475290	GTP-binding protein	*Cicer arietinum*	cDNA clone	Experimental	NA
1	1	10	3	DY475447	Protein transport protein	*Cicer arietinum*	cDNA clone	Experimental	NA
1	1	10	4	DY475488	DNAJ-like protein	*Cicer arietinum*	cDNA clone	Experimental	NA
1	1	10	5	DY475523	Sorting nexin protein	*Cicer arietinum*	cDNA clone	Experimental	NA
1	1	10	6	DY475065	Unclear	*Cicer arietinum*	cDNA clone	Experimental	NA
1	1	10	7	DY475086	Unclear	*Cicer arietinum*	cDNA clone	Experimental	NA
1	1	10	8	DY475097	Unclear	*Cicer arietinum*	cDNA clone	Experimental	NA
1	1	10	9	DY475259	Unclear	*Cicer arietinum*	cDNA clone	Experimental	NA
1	1	10	10	DY475264	Unclear	*Cicer arietinum*	cDNA clone	Experimental	NA
1	1	10	11	DY475272	Unclear	*Cicer arietinum*	cDNA clone	Experimental	NA
1	1	10	12	DY475274	Unclear	*Cicer arietinum*	cDNA clone	Experimental	NA
1	1	10	13	DY475292	Unclear	*Cicer arietinum*	cDNA clone	Experimental	NA
1	1	10	14	DY475319	Unclear	*Cicer arietinum*	cDNA clone	Experimental	NA
1	1	11	1	DY475489	Chlorophyll a/b binding protein	*Cicer arietinum*	cDNA clone	Experimental	NA
1	1	11	2	DY475518	Chloroplast DNA between the RUBISCO large subunit and ATPase (beta) genes	*Cicer arietinum*	cDNA clone	Experimental	NA
1	1	11	3	DY475063	Chloroplast 30S ribosomal protein S12	*Cicer arietinum*	cDNA clone	Experimental	NA
1	1	11	4	DY475104	Ribosomal protein L41	*Cicer arietinum*	cDNA clone	Experimental	NA
1	1	11	5	DY475117	40S ribosomal protein S15	*Cicer arietinum*	cDNA clone	Experimental	NA

1	1	11	6	DY475122	Amino acid transferase	*Cicer arietinum*	cDNA clone	Experimental	NA
1	1	11	7	DY475420	26S ribosomal protein	*Cicer arietinum*	cDNA clone	Experimental	NA
1	1	11	8	DY475425	60S ribosomal protein L23	*Cicer arietinum*	cDNA clone	Experimental	NA
1	1	11	9	DY475442	Translation initiation factor	*Cicer arietinum*	cDNA clone	Experimental	NA
1	1	11	10	DY475499	S28 ribosomal protein	*Cicer arietinum*	cDNA clone	Experimental	NA
1	1	11	11	DY475506	Chloroplast 50S ribosomal protein	*Cicer arietinum*	cDNA clone	Experimental	NA
1	1	11	12	DY475510	30S ribosomal protein S13	*Cicer arietinum*	cDNA clone	Experimental	NA
1	1	11	13	DY475524	40S ribosomal protein S27	*Cicer arietinum*	cDNA clone	Experimental	NA
1	1	11	14	DY475101	Chloroplast 16S rRNA	*Cicer arietinum*	cDNA clone	Experimental	NA
1	1	12	1	DY475500	Zinc-binding dehydrogenase	*Cicer arietinum*	cDNA clone	Experimental	NA
1	1	12	2	DY475530	Thiamine biosynthesis protein	*Cicer arietinum*	cDNA clone	Experimental	NA
1	1	12	3	CV793610	Class 10 pathogenesis related protein	*Cicer arietinum*	cDNA clone	Experimental	NA
1	1	12	4	CV793594	Transcription factor of the AP2/EREBP1 DNA binding domain	*Cicer arietinum*	cDNA clone	Experimental	NA
1	1	12	5	DY475047	Photosystem I reaction centre subunit IX	*Cicer arietinum*	cDNA clone	Experimental	NA
1	1	12	6	DY475058	Chlorplast CP12 mRNA	*Cicer arietinum*	cDNA clone	Experimental	NA
1	1	12	7	DY475069	Thioredoxin	*Cicer arietinum*	cDNA clone	Experimental	NA
1	1	12	8	DY475083	Ferredoxin-NADP reductase (EC 1.18.1.2)	*Cicer arietinum*	cDNA clone	Experimental	NA
1	1	12	9	DY475128	Photosystem I reaction centre subunit IV	*Cicer arietinum*	cDNA clone	Experimental	NA
1	1	12	10	DY475132	Photosystem I reaction centre subunit XI	*Cicer arietinum*	cDNA clone	Experimental	NA
1	1	12	11	DY475142	Photosystem II D2 protein	*Cicer arietinum*	cDNA clone	Experimental	NA
1	1	12	12	DY475148	Photosystem II protein	*Cicer arietinum*	cDNA clone	Experimental	NA
1	1	12	13	DY475454	Chlorophyll a/b binding protein	*Cicer arietinum*	cDNA clone	Experimental	NA
1	1	12	14	DY475480	Photosystem II core complex protein psbY	*Cicer arietinum*	cDNA clone	Experimental	NA
1	1	13	1	DY475379	Thymidylate kinase	*Cicer arietinum*	cDNA clone	Experimental	NA
1	1	13	2	DY475550	WD repeat protein	*Cicer arietinum*	cDNA clone	Experimental	NA
1	1	13	3	DY475155	Superoxide dismutase (EC 1.15.1.1)	*Cicer arietinum*	cDNA clone	Experimental	NA
1	1	13	4	DY475179	Acetyl transferase	*Cicer arietinum*	cDNA clone	Experimental	NA
1	1	13	5	DY475181	Apocytochrome F	*Cicer arietinum*	cDNA clone	Experimental	NA
1	1	13	6	DY475199	Squalene epoxidase enzyme (EC 1.14.99.7)	*Cicer arietinum*	cDNA clone	Experimental	NA
1	1	13	7	DY475212	Glyceraldehyde 3-phosphate dehydrogenase (EC 1.2.1.12)	*Cicer arietinum*	cDNA clone	Experimental	NA
1	1	13	8	DY475240	Ribose 5-phosphate isomerase (EC 5.3.1.6)	*Cicer arietinum*	cDNA clone	Experimental	NA
1	1	13	9	DY475234	Glycine cleavage system H protein	*Cicer arietinum*	cDNA clone	Experimental	NA
1	1	13	10	DY475547	Fructose-1,6-bisphosphate aldolase (EC 4.1.2.13)	*Cicer arietinum*	cDNA clone	Experimental	NA
1	1	13	11	DY475443	Succinate dehydrogenase subunit 3	*Cicer arietinum*	cDNA clone	Experimental	NA
1	1	13	12	DY475457	Lipoamide dehydrogenase (EC 1.8.1.4)	*Cicer arietinum*	cDNA clone	Experimental	NA

1	1	13	13	DY475475	Asparagine synthetase (EC 6.3.5.4)	*Cicer arietinum*	cDNA clone	Experimental	NA
1	1	13	14	DY475551	Homogentisate 1,2 dioxygenase (EC 1.13.11.5)	*Cicer arietinum*	cDNA clone	Experimental	NA
1	1	14	1	DY475112	Nucleotide-sugar epimerase	*Cicer arietinum*	cDNA clone	Experimental	NA
1	1	14	2	DY475244	Nucleotide-sugar dehydratase	*Cicer arietinum*	cDNA clone	Experimental	NA
1	1	14	3	DY475300	Actin	*Cicer arietinum*	cDNA clone	Experimental	NA
1	1	14	4	DY475372	Adenosylhomocysteinase	*Cicer arietinum*	cDNA clone	Experimental	NA
1	1	14	5	DY475049	Metallothionein protein (MT-2)	*Cicer arietinum*	cDNA clone	Experimental	NA
1	1	14	6	DY475076	Phosphate-induced protein	*Cicer arietinum*	cDNA clone	Experimental	NA
1	1	14	7	DY475092	Dehydrin cold-induced protein	*Cicer arietinum*	cDNA clone	Experimental	NA
1	1	14	8	DY475137	Auxin repressed protein	*Cicer arietinum*	cDNA clone	Experimental	NA
1	1	14	9	DY475509	PPF1 - post floral protein	*Cicer arietinum*	cDNA clone	Experimental	NA
1	1	14	10	DY475077	Protein kinase	*Cicer arietinum*	cDNA clone	Experimental	NA
1	1	14	11	DY475103	Protein kinase	*Cicer arietinum*	cDNA clone	Experimental	NA
1	1	14	12	DY475198	SNAP25 protein	*Cicer arietinum*	cDNA clone	Experimental	NA
1	1	14	13	DY475248	Polymorphic antigen membrane protein	*Cicer arietinum*	cDNA clone	Experimental	NA
1	1	14	14	DY475320	Serine/threonine protein kinase	*Cicer arietinum*	cDNA clone	Experimental	NA
1	2	1	1	NA	NBS-LRR putative RGA Aj516078	*Lens culinaris* (ILL7537)	Genomic PCR product	Experimental	NA
1	2	1	2	NA	NBS-LRR putative RGA Aj516084	*Lens culinaris* (ILL7537)	Genomic PCR product	Experimental	NA
1	2	1	3	NA	NBS-LRR putative RGA Aj516078	*Lens culinaris* (ILL6002)	Genomic PCR product	Experimental	NA
1	2	1	4	NA	NBS-LRR putative RGA Aj516083	*Lens culinaris* (ILL6002)	Genomic PCR product	Experimental	NA
1	2	1	5	NA	NBS-LRR putative RGA Aj516088	*Lens culinaris* (ILL6002)	Genomic PCR product	Experimental	NA
1	2	1	6	NA	Copper amine oxidase	*Lens culinaris* (ILL6002)	Genomic PCR product	Experimental	NA
1	2	1	7	NA	Isoflavone synthase	*Lens culinaris* (ILL6002)	Genomic PCR product	Experimental	NA
1	2	1	8	NA	Printing Control	NA	Oligo	Control	Printing
1	2	1	9	NA	Blank	NA	Blank	Control	Negative
1	2	1	10	NA	Blank	NA	Blank	Control	Negative
1	2	2	1	DY396406	Metallothionein-like protein 1	*Lathyrus sativus*	cDNA clone	Experimental	NA
1	2	2	2	DY396402	Alpha-amylase	*Lathyrus sativus*	cDNA clone	Experimental	NA
1	2	2	3	DY396399	Cornifin alpha (small proline-rich protein 1)	*Lathyrus sativus*	cDNA clone	Experimental	NA
1	2	2	4	DY396270	Putative deoxycytidylate deaminase	*Lathyrus sativus*	cDNA clone	Experimental	NA
1	2	2	5	DY396267	Enolase	*Lathyrus sativus*	cDNA clone	Experimental	NA
1	2	2	6	DY396428	Polyubiquitin	*Lathyrus sativus*	cDNA clone	Experimental	NA
1	2	2	7	DY396420	Similarity to heat shock related protein	*Lathyrus sativus*	cDNA clone	Experimental	NA
1	2	2	8	DY396317	Putative glutaredoxin	*Lathyrus sativus*	cDNA clone	Experimental	NA
1	2	2	9	DY396419	Putative tonoplast intrinsic protein	*Lathyrus sativus*	cDNA clone	Experimental	NA
1	2	2	10	NA	NBS-LRR putative RGA Aj516061	*Lens culinaris (*ILL7537)	Genomic PCR product	Experimental	NA

1	2	2	11	NA	NBS-LRR putative RGA Aj516064	*Lens culinaris* (ILL7537)	Genomic PCR product	Experimental	NA
1	2	2	12	NA	NBS-LRR putative RGA Aj516067	*Lens culinaris* (ILL7537)	Genomic PCR product	Experimental	NA
1	2	2	13	NA	NBS-LRR putative RGA Aj516071	*Lens culinaris* (ILL7537)	Genomic PCR product	Experimental	NA
1	2	2	14	NA	NBS-LRR putative RGA Aj516073	*Lens culinaris* (ILL7537)	Genomic PCR product	Experimental	NA
1	2	3	1	DY396411	Calmodulin-binding protein/ER66	*Lathyrus sativus*	cDNA clone	Experimental	NA
1	2	3	2	DY396416	Disease resistance response protein 230 precursor	*Lathyrus sativus*	cDNA clone	Experimental	NA
1	2	3	3	DY396422	Protein kinase-like protein	*Lathyrus sativus*	cDNA clone	Experimental	NA
1	2	3	4	DY396426	Subtilisin inhibitors I and II (ASI-I and ASI-II)	*Lathyrus sativus*	cDNA clone	Experimental	NA
1	2	3	5	DY396427	Lectin-like protein	*Lathyrus sativus*	cDNA clone	Experimental	NA
1	2	3	6	DY396430	Chalcone reductase	*Lathyrus sativus*	cDNA clone	Experimental	NA
1	2	3	7	DY396310	Polyubiquitin	*Lathyrus sativus*	cDNA clone	Experimental	NA
1	2	3	8	DY396306	Epoxide hydrolase	*Lathyrus sativus*	cDNA clone	Experimental	NA
1	2	3	9	DY396342	Glycine-rich cell wall protein GRP 1.8	*Lathyrus sativus*	cDNA clone	Experimental	NA
1	2	3	10	DY396340	Cytochrome B5	*Lathyrus sativus*	cDNA clone	Experimental	NA
1	2	3	11	DY396337	Alpha-amylase precursor	*Lathyrus sativus*	cDNA clone	Experimental	NA
1	2	3	12	DY396326	Ubiquitin	*Lathyrus sativus*	cDNA clone	Experimental	NA
1	2	3	13	DY396368	Ubiquitin	*Lathyrus sativus*	cDNA clone	Experimental	NA
1	2	3	14	DY396363	Magnesium chelatase subunit	*Lathyrus sativus*	cDNA clone	Experimental	NA
1	2	4	1	DY396262	Probable Ca-binding mitochondrial carrier AT2G35800	*Lathyrus sativus*	cDNA clone	Experimental	NA
1	2	4	2	DY396265	Disease resistance response protein DRRG49-C	*Lathyrus sativus*	cDNA clone	Experimental	NA
1	2	4	3	DY396275	Putative chitinase	*Lathyrus sativus*	cDNA clone	Experimental	NA
1	2	4	4	DY396277	Disease resistance response protein 39 precursor	*Lathyrus sativus*	cDNA clone	Experimental	NA
1	2	4	5	DY396314	Immunophilin	*Lathyrus sativus*	cDNA clone	Experimental	NA
1	2	4	6	DY396331	Glutathione peroxidase	*Lathyrus sativus*	cDNA clone	Experimental	NA
1	2	4	7	DY396335	Nitrate transporter NRT1-1	*Lathyrus sativus*	cDNA clone	Experimental	NA
1	2	4	8	DY396436	Putative nuclear transport factor 2	*Lathyrus sativus*	cDNA clone	Experimental	NA
1	2	4	9	DY396345	Protein kinase-like protein (Serine/Threonine kinase PBS1)	*Lathyrus sativus*	cDNA clone	Experimental	NA
1	2	4	10	DY396351	Putative protein kinase	*Lathyrus sativus*	cDNA clone	Experimental	NA
1	2	4	11	DY396358	Laccase-like protein	*Lathyrus sativus*	cDNA clone	Experimental	NA
1	2	4	12	DY396362	Protein kinase-like protein	*Lathyrus sativus*	cDNA clone	Experimental	NA
1	2	4	13	DY396395	EREBP-4	*Lathyrus sativus*	cDNA clone	Experimental	NA
1	2	4	14	DY396384	Pathogenesis-related protein 4A	*Lathyrus sativus*	cDNA clone	Experimental	NA
1	2	5	1	NA	Unknown	*Cicer arietinum*	cDNA clone	Experimental	NA
1	2	5	2	EB085037	Unknown	*Cicer arietinum*	cDNA clone	Experimental	NA
1	2	5	3	EB085046	Unclear	*Cicer arietinum*	cDNA clone	Experimental	NA

1	2	5	4	NA	Unknown	*Cicer arietinum*	cDNA clone	Experimental	NA
1	2	5	5	NA	Unknown	*Cicer arietinum*	cDNA clone	Experimental	NA
1	2	5	6	NA	Unknown	*Cicer arietinum*	cDNA clone	Experimental	NA
1	2	5	7	DY475539	Unknown	*Cicer arietinum*	cDNA clone	Experimental	NA
1	2	5	8	NA	Unknown	*Cicer arietinum*	cDNA clone	Experimental	NA
1	2	5	9	EB085050	Chloroplast DNA	*Cicer arietinum*	cDNA clone	Experimental	NA
1	2	5	10	NA	Unknown	*Cicer arietinum*	cDNA clone	Experimental	NA
1	2	5	11	NA	Normalisation control 2	*Cicer arietinum*	cDNA clone	Control	Normalisation
1	2	5	12	NA	Digested pGEM-T Easy Vector II (Promega) Plasmid *AluI*	NA	Digested Plasmid	Control	Negative
1	2	5	13	NA	SMART (Clontech) PCR primer	NA	Oligo	Control	Negative
1	2	5	14	DY396260	Subtilisin Inhibitors I and II (ASI-I and ASI-I)	*Lathyrus sativus*	cDNA clone	Experimental	NA
1	2	6	1	EB085028	Unknown	*Cicer arietinum*	cDNA clone	Experimental	NA
1	2	6	2	EB085029	Unknown	*Cicer arietinum*	cDNA clone	Experimental	NA
1	2	6	3	EB085030	Unknown	*Cicer arietinum*	cDNA clone	Experimental	NA
1	2	6	4	NA	Unknown	*Cicer arietinum*	cDNA clone	Experimental	NA
1	2	6	5	NA	Unknown	*Cicer arietinum*	cDNA clone	Experimental	NA
1	2	6	6	NA	Unknown	*Cicer arietinum*	cDNA clone	Experimental	NA
1	2	6	7	NA	Unknown	*Cicer arietinum*	cDNA clone	Experimental	NA
1	2	6	8	EB085032	Disease resistance response protein DRRG49-C	*Cicer arietinum*	cDNA clone	Experimental	NA
1	2	6	9	EB085023	60S rRNA	*Cicer arietinum*	cDNA clone	Experimental	NA
1	2	6	10	DY475533	Unknown	*Cicer arietinum*	cDNA clone	Experimental	NA
1	2	6	11	EB085061	Unknown	*Cicer arietinum*	cDNA clone	Experimental	NA
1	2	6	12	NA	Unknown	*Cicer arietinum*	cDNA clone	Experimental	NA
1	2	6	13	EB085026	Unknown	*Cicer arietinum*	cDNA clone	Experimental	NA
1	2	6	14	NA	Unknown	*Cicer arietinum*	cDNA clone	Experimental	NA
1	2	7	1	DY475391	Unknown	*Cicer arietinum*	cDNA clone	Experimental	NA
1	2	7	2	DY475399	Unknown	*Cicer arietinum*	cDNA clone	Experimental	NA
1	2	7	3	DY475407	Unknown	*Cicer arietinum*	cDNA clone	Experimental	NA
1	2	7	4	DY475414	Unknown	*Cicer arietinum*	cDNA clone	Experimental	NA
1	2	7	5	DY475426	Unknown	*Cicer arietinum*	cDNA clone	Experimental	NA
1	2	7	6	DY475431	Unknown	*Cicer arietinum*	cDNA clone	Experimental	NA
1	2	7	7	DY475485	Unknown	*Cicer arietinum*	cDNA clone	Experimental	NA
1	2	7	8	DY475491	Unknown	*Cicer arietinum*	cDNA clone	Experimental	NA
1	2	7	9	DY475553	Unknown	*Cicer arietinum*	cDNA clone	Experimental	NA
1	2	7	10	DY475519	Unknown	*Cicer arietinum*	cDNA clone	Experimental	NA
1	2	7	11	DY475521	Unknown	*Cicer arietinum*	cDNA clone	Experimental	NA

1	2	7	12	NA	Unknown	*Cicer arietinum*	cDNA clone	Experimental	NA
1	2	7	13	EB085014	Unknown	*Cicer arietinum*	cDNA clone	Experimental	NA
1	2	7	14	NA	Unknown	*Cicer arietinum*	cDNA clone	Experimental	NA
1	2	8	1	DY475230	Unknown	*Cicer arietinum*	cDNA clone	Experimental	NA
1	2	8	2	DY475236	Unknown	*Cicer arietinum*	cDNA clone	Experimental	NA
1	2	8	3	DY475243	Unknown	*Cicer arietinum*	cDNA clone	Experimental	NA
1	2	8	4	DY475255	Unknown	*Cicer arietinum*	cDNA clone	Experimental	NA
1	2	8	5	DY475298	Unknown	*Cicer arietinum*	cDNA clone	Experimental	NA
1	2	8	6	DY475303	Unknown	*Cicer arietinum*	cDNA clone	Experimental	NA
1	2	8	7	DY475311	Unknown	*Cicer arietinum*	cDNA clone	Experimental	NA
1	2	8	8	DY475315	Unknown	*Cicer arietinum*	cDNA clone	Experimental	NA
1	2	8	9	DY475327	Unknown	*Cicer arietinum*	cDNA clone	Experimental	NA
1	2	8	10	DY475331	Unknown	*Cicer arietinum*	cDNA clone	Experimental	NA
1	2	8	11	DY475337	Unknown	*Cicer arietinum*	cDNA clone	Experimental	NA
1	2	8	12	DY475339	Unknown	*Cicer arietinum*	cDNA clone	Experimental	NA
1	2	8	13	DY475373	Unknown	*Cicer arietinum*	cDNA clone	Experimental	NA
1	2	8	14	DY475382	Unknown	*Cicer arietinum*	cDNA clone	Experimental	NA
1	2	9	1	DY475444	Unclear	*Cicer arietinum*	cDNA clone	Experimental	NA
1	2	9	2	DY475473	Unclear	*Cicer arietinum*	cDNA clone	Experimental	NA
1	2	9	3	DY475081	Unknown	*Cicer arietinum*	cDNA clone	Experimental	NA
1	2	9	4	DY475085	Unknown	*Cicer arietinum*	cDNA clone	Experimental	NA
1	2	9	5	DY475094	Unknown	*Cicer arietinum*	cDNA clone	Experimental	NA
1	2	9	6	DY475100	Unknown	*Cicer arietinum*	cDNA clone	Experimental	NA
1	2	9	7	DY475106	Unknown	*Cicer arietinum*	cDNA clone	Experimental	NA
1	2	9	8	DY475125	Unknown	*Cicer arietinum*	cDNA clone	Experimental	NA
1	2	9	9	DY475133	Unknown	*Cicer arietinum*	cDNA clone	Experimental	NA
1	2	9	10	DY475051	Unknown	*Cicer arietinum*	cDNA clone	Experimental	NA
1	2	9	11	DY475203	Unknown	*Cicer arietinum*	cDNA clone	Experimental	NA
1	2	9	12	DY475208	Unknown	*Cicer arietinum*	cDNA clone	Experimental	NA
1	2	9	13	DY475215	Unknown	*Cicer arietinum*	cDNA clone	Experimental	NA
1	2	9	14	DY475219	Unknown	*Cicer arietinum*	cDNA clone	Experimental	NA
1	2	10	1	DY475114	Unclear	*Cicer arietinum*	cDNA clone	Experimental	NA
1	2	10	2	DY475126	Unclear	*Cicer arietinum*	cDNA clone	Experimental	NA
1	2	10	3	DY475175	Unclear	*Cicer arietinum*	cDNA clone	Experimental	NA
1	2	10	4	DY475205	Unclear	*Cicer arietinum*	cDNA clone	Experimental	NA
1	2	10	5	DY475217	Unclear	*Cicer arietinum*	cDNA clone	Experimental	NA

1	2	10	6	DY475222	Unclear	*Cicer arietinum*	cDNA clone	Experimental	NA
1	2	10	7	DY475226	Unclear	*Cicer arietinum*	cDNA clone	Experimental	NA
1	2	10	8	DY475235	Unclear	*Cicer arietinum*	cDNA clone	Experimental	NA
1	2	10	9	DY475367	Unclear	*Cicer arietinum*	cDNA clone	Experimental	NA
1	2	10	10	DY475380	Unclear	*Cicer arietinum*	cDNA clone	Experimental	NA
1	2	10	11	DY475388	Unclear	*Cicer arietinum*	cDNA clone	Experimental	NA
1	2	10	12	DY475549	Unclear	*Cicer arietinum*	cDNA clone	Experimental	NA
1	2	10	13	DY475409	Unclear	*Cicer arietinum*	cDNA clone	Experimental	NA
1	2	10	14	DY475418	Unclear	*Cicer arietinum*	cDNA clone	Experimental	NA
1	2	11	1	DY475312	60S ribosomal protein L14	*Cicer arietinum*	cDNA clone	Experimental	NA
1	2	11	2	DY475324	60S ribosomal protein L19	*Cicer arietinum*	cDNA clone	Experimental	NA
1	2	11	3	DY475344	Chloroplast 50S ribosomal protein L14	*Cicer arietinum*	cDNA clone	Experimental	NA
1	2	11	4	DY475354	40S ribosomal protein S27A	*Cicer arietinum*	cDNA clone	Experimental	NA
1	2	11	5	DY475371	60S ribosomal protein L38	*Cicer arietinum*	cDNA clone	Experimental	NA
1	2	11	6	DY475395	60S ribosomal protein L11	*Cicer arietinum*	cDNA clone	Experimental	NA
1	2	11	7	DY475109	Mitochondrial 26S rRNA	*Cicer arietinum*	cDNA clone	Experimental	NA
1	2	11	8	DY475146	Chloroplast 16S rRNA	*Cicer arietinum*	cDNA clone	Experimental	NA
1	2	11	9	DY475153	26S ribosomal RNA	*Cicer arietinum*	cDNA clone	Experimental	NA
1	2	11	10	DY475196	RNA polymerase beta subunit	*Cicer arietinum*	cDNA clone	Experimental	NA
1	2	11	11	DY475297	RNA binding protein	*Cicer arietinum*	cDNA clone	Experimental	NA
1	2	11	12	DY475419	DNA directed RNA polymerase	*Cicer arietinum*	cDNA clone	Experimental	NA
1	2	11	13	DY475074	Protein transport protein	*Cicer arietinum*	cDNA clone	Experimental	NA
1	2	11	14	DY475169	Potassium channel regulatory factor	*Cicer arietinum*	cDNA clone	Experimental	NA
1	2	12	1	CV793606	SNAKIN2 antimicrobial peptide precursor	*Cicer arietinum*	cDNA clone	Experimental	NA
1	2	12	2	CV793608	SNAKIN2 antimicrobial peptide precursor	*Cicer arietinum*	cDNA clone	Experimental	NA
1	2	12	3	CV793603	Nematode resistance protein *Hs1pro-1* homolog	*Cicer arietinum*	cDNA clone	Experimental	NA
1	2	12	4	CV793587	Extensin-like protein	*Cicer arietinum*	cDNA clone	Experimental	NA
1	2	12	5	DY475163	Chlorophyll a/b binding protein	*Cicer arietinum*	cDNA clone	Experimental	NA
1	2	12	6	DY475202	Chlorophyll a/b binding protein	*Cicer arietinum*	cDNA clone	Experimental	NA
1	2	12	7	DY475245	ATP synthase (EC 3.6.1.34)	*Cicer arietinum*	cDNA clone	Experimental	NA
1	2	12	8	DY475287	NADH-plastoquinone oxidoreductase subunit I (EC 1.6.5.3)	*Cicer arietinum*	cDNA clone	Experimental	NA
1	2	12	9	DY475304	Similar to ferredoxin-thioredoxin reductase	*Cicer arietinum*	cDNA clone	Experimental	NA
1	2	12	10	DY475316	NADH dehydrogenase	*Cicer arietinum*	cDNA clone	Experimental	NA
1	2	12	11	DY475402	Chloroplast DNA	*Cicer arietinum*	cDNA clone	Experimental	NA
1	2	12	12	DY475430	Chlorophyll a/b binding protein	*Cicer arietinum*	cDNA clone	Experimental	NA
1	2	12	13	DY475131	50S ribosomal protein L12	*Cicer arietinum*	cDNA clone	Experimental	NA

1	2	12	14	DY475238	Chloroplast 30S ribosomal protein S7	*Cicer arietinum*	cDNA clone	Experimental	NA
1	2	13	1	DY475136	Cytochrome P450	*Cicer arietinum*	cDNA clone	Experimental	NA
1	2	13	2	DY475149	UDP-glucose 4-epimerase (EC 5.1.3.2)	*Cicer arietinum*	cDNA clone	Experimental	NA
1	2	13	3	DY475286	Similar to alpha galactosidase	*Cicer arietinum*	cDNA clone	Experimental	NA
1	2	13	4	DY475306	Cationic peroxidase (EC 1.11.1.7)	*Cicer arietinum*	cDNA clone	Experimental	NA
1	2	13	5	DY475309	Xylose isomerase (EC 5.3.1.5)	*Cicer arietinum*	cDNA clone	Experimental	NA
1	2	13	6	DY475374	Cytochrome P450	*Cicer arietinum*	cDNA clone	Experimental	NA
1	2	13	7	DY475387	Peptidase-like protein	*Cicer arietinum*	cDNA clone	Experimental	NA
1	2	13	8	DY475396	Similar to endopeptidase	*Cicer arietinum*	cDNA clone	Experimental	NA
1	2	13	9	DY475403	Carbonic anhydrase like protein (EC 4.2.1.1)	*Cicer arietinum*	cDNA clone	Experimental	NA
1	2	13	10	DY475415	Beta glucosidase (EC 3.2.1.21)	*Cicer arietinum*	cDNA clone	Experimental	NA
1	2	13	11	CV793593	Homology to putative disease resistance protein from *A.thaliana*	*Cicer arietinum*	cDNA clone	Experimental	NA
1	2	13	12	CV793598	beta-1,3-glucanase	*Cicer arietinum*	cDNA clone	Experimental	NA
1	2	13	13	CV793600	Transcriptional activator	*Cicer arietinum*	cDNA clone	Experimental	NA
1	2	13	14	CV793602	Cinnamyl-alcohol-dehydrogenase	*Cicer arietinum*	cDNA clone	Experimental	NA
1	2	14	1	DY475172	Phosphate-induced protein	*Cicer arietinum*	cDNA clone	Experimental	NA
1	2	14	2	DY475192	Dehydration-induced protein	*Cicer arietinum*	cDNA clone	Experimental	NA
1	2	14	3	DY475220	Wound-induced protein	*Cicer arietinum*	cDNA clone	Experimental	NA
1	2	14	4	DY475237	Translation initiation factor	*Cicer arietinum*	cDNA clone	Experimental	NA
1	2	14	5	DY475254	Wound-induced protein	*Cicer arietinum*	cDNA clone	Experimental	NA
1	2	14	6	DY475278	Heat shock protein	*Cicer arietinum*	cDNA clone	Experimental	NA
1	2	14	7	DY475335	Heat shock protein	*Cicer arietinum*	cDNA clone	Experimental	NA
1	2	14	8	DY475453	Heat shock protein	*Cicer arietinum*	cDNA clone	Experimental	NA
1	2	14	9	DY475463	Similarity to protein-tyrosine-kinase receptor (EC 2.7.1.112)	*Cicer arietinum*	cDNA clone	Experimental	NA
1	2	14	10	DY475478	Hypothetical transmembrane protein	*Cicer arietinum*	cDNA clone	Experimental	NA
1	2	14	11	DY475525	Actin regulating protein	*Cicer arietinum*	cDNA clone	Experimental	NA
1	2	14	12	DY475068	L-allo-threonine aldolase (EC 4.1.2.5)	*Cicer arietinum*	cDNA clone	Experimental	NA
1	2	14	13	DY475105	Sucrose synthase (EC 2.4.1.14)	*Cicer arietinum*	cDNA clone	Experimental	NA
1	2	14	14	DY475108	Asparagine synthetase (EC 6.3.5.4)	*Cicer arietinum*	cDNA clone	Experimental	NA
1	3	1	1	DY396330	Thrioredoxin H-type 1	*Lathyrus sativus*	cDNA clone	Experimental	NA
1	3	1	2	DY396404	Glutathione S-transferase GST 8	*Lathyrus sativus*	cDNA clone	Experimental	NA
1	3	1	3	NA	Copper amine oxidase	*Lens culinaris* (ILL7537)	Genomic PCR product	Experimental	NA
1	3	1	4	NA	Isoflavone synthase	*Lens culinaris* (ILL7537)	Genomic PCR product	Experimental	NA
1	3	1	5	NA	NBS-LRR putative RGA Aj516060	*Lens culinaris* (ILL6002)	Genomic PCR product	Experimental	NA
1	3	1	6	NA	NBS-LRR putative RGA Aj516062	*Lens culinaris* (ILL6002)	Genomic PCR product	Experimental	NA

1	3	1	7	NA	NBS-LRR putative RGA Aj516064	*Lens culinaris* (ILL6002)	Genomic PCR product	Experimental	NA
1	3	1	8	NA	NBS-LRR putative RGA Aj516067	*Lens culinaris* (ILL6002)	Genomic PCR product	Experimental	NA
1	3	1	9	NA	NBS-LRR putative RGA Aj516072	*Lens culinaris* (ILL6002)	Genomic PCR product	Experimental	NA
1	3	1	10	NA	NBS-LRR putative RGA Aj516076	*Lens culinaris* (ILL6002)	Genomic PCR product	Experimental	NA
1	3	2	1	DY396354	Polyubiquitin	*Lathyrus sativus*	cDNA clone	Experimental	NA
1	3	2	2	DY396413	Catalase	*Lathyrus sativus*	cDNA clone	Experimental	NA
1	3	2	3	DY396383	Putative extracelluler dermal glycoprotein	*Lathyrus sativus*	cDNA clone	Experimental	NA
1	3	2	4	DY396377	Ripening-related protein	*Lathyrus sativus*	cDNA clone	Experimental	NA
1	3	2	5	DY396373	Metallothionein-like protein 1	*Lathyrus sativus*	cDNA clone	Experimental	NA
1	3	2	6	DY396370	Ubiquitin-conjugating enzyme E2	*Lathyrus sativus*	cDNA clone	Experimental	NA
1	3	2	7	DY396412	Poly(A)-binding protein	*Lathyrus sativus*	cDNA clone	Experimental	NA
1	3	2	8	DY396408	Ubiquitin-specific protease 16	*Lathyrus sativus*	cDNA clone	Experimental	NA
1	3	2	9	DY396284	Histone deacetylase 2 isoform B	*Lathyrus sativus*	cDNA clone	Experimental	NA
1	3	2	10	DY396287	Kinesin-like protein	*Lathyrus sativus*	cDNA clone	Experimental	NA
1	3	2	11	DY396280	Serine carboxypeptidase isolag	*Lathyrus sativus*	cDNA clone	Experimental	NA
1	3	2	12	DY396396	Cysteine proteinase 15A precursor	*Lathyrus sativus*	cDNA clone	Experimental	NA
1	3	2	13	DY396348	Glycolate oxidase	*Lathyrus sativus*	cDNA clone	Experimental	NA
1	3	2	14	DY396435	L-ascorbate peroxidase cytosolic	*Lathyrus sativus*	cDNA clone	Experimental	NA
1	3	3	1	DY396375	Putative protein kinase	*Lathyrus sativus*	cDNA clone	Experimental	NA
1	3	3	2	DY396381	Small GTP-binding protein	*Lathyrus sativus*	cDNA clone	Experimental	NA
1	3	3	3	DY396388	Pathogenesis-related protein 4A	*Lathyrus sativus*	cDNA clone	Experimental	NA
1	3	3	4	DY396385	TMV resistance protein-like	*Lathyrus sativus*	cDNA clone	Experimental	NA
1	3	3	5	DY396390	Disease resistance response protein 230 (DRR230-a)	*Lathyrus sativus*	cDNA clone	Experimental	NA
1	3	3	6	DY396393	6-Phosphogluconate dehydrogenase	*Lathyrus sativus*	cDNA clone	Experimental	NA
1	3	3	7	DY396303	Ubiquitin-like protein	*Lathyrus sativus*	cDNA clone	Experimental	NA
1	3	3	8	DY396298	Environmental stress inducible protein	*Lathyrus sativus*	cDNA clone	Experimental	NA
1	3	3	9	DY396295	Metallothionein-like protein	*Lathyrus sativus*	cDNA clone	Experimental	NA
1	3	3	10	DY396278	Ubiquitin	*Lathyrus sativus*	cDNA clone	Experimental	NA
1	3	3	11	DY396263	Transcription factor NTLIM1	*Lathyrus sativus*	cDNA clone	Experimental	NA
1	3	3	12	DY396321	Dehydration stress-induced protein	*Lathyrus sativus*	cDNA clone	Experimental	NA
1	3	3	13	DY396319	Polyubiquitin	*Lathyrus sativus*	cDNA clone	Experimental	NA
1	3	3	14	DY396315	Auxin-responsive protein IAA9	*Lathyrus sativus*	cDNA clone	Experimental	NA
1	3	4	1	DY475542	18S rRNA	*Cicer arietinum*	cDNA clone	Experimental	NA
1	3	4	2	EB085056	Beta-galactosidase (EC 3.2.1.23)	*Cicer arietinum*	cDNA clone	Experimental	NA
1	3	4	3	EB085057	Unknown	*Cicer arietinum*	cDNA clone	Experimental	NA
1	3	4	4	NA	Normalisation control 1	*Cicer arietinum*	cDNA clone	Control	Normalisation

1	3	4	5	DY396285	Protein kinase C inhibitor-like protein	*Lathyrus sativus*	cDNA clone	Experimental	NA
1	3	4	6	DY396291	Putative ARF1 GTPase activating protein	*Lathyrus sativus*	cDNA clone	Experimental	NA
1	3	4	7	DY396294	Putative steroid binding protein	*Lathyrus sativus*	cDNA clone	Experimental	NA
1	3	4	8	DY396297	Isovaleryl-coa dehydrogenase	*Lathyrus sativus*	cDNA clone	Experimental	NA
1	3	4	9	DY396300	ATHP3 (histidine-containing phosphotransfer protein like)	*Lathyrus sativus*	cDNA clone	Experimental	NA
1	3	4	10	DY396304	Putative steroid binding protein	*Lathyrus sativus*	cDNA clone	Experimental	NA
1	3	4	11	DY396307	Serine/thrionine protein kinase	*Lathyrus sativus*	cDNA clone	Experimental	NA
1	3	4	12	DY396313	Guanine nucleotide regulatory protein	*Lathyrus sativus*	cDNA clone	Experimental	NA
1	3	4	13	DY396367	Small GTP-binding protein	*Lathyrus sativus*	cDNA clone	Experimental	NA
1	3	4	14	DY396372	Pathogenesis-related protein 4A	*Lathyrus sativus*	cDNA clone	Experimental	NA
1	3	5	1	EB085020	Unknown	*Cicer arietinum*	cDNA clone	Experimental	NA
1	3	5	2	EB085022	Unknown	*Cicer arietinum*	cDNA clone	Experimental	NA
1	3	5	3	NA	Unknown	*Cicer arietinum*	cDNA clone	Experimental	NA
1	3	5	4	EB085040	Unknown	*Cicer arietinum*	cDNA clone	Experimental	NA
1	3	5	5	NA	Unknown	*Cicer arietinum*	cDNA clone	Experimental	NA
1	3	5	6	EB085041	Unknown	*Cicer arietinum*	cDNA clone	Experimental	NA
1	3	5	7	EB085042	Phosphate-induced protein	*Cicer arietinum*	cDNA clone	Experimental	NA
1	3	5	8	EB085044	Unknown	*Cicer arietinum*	cDNA clone	Experimental	NA
1	3	5	9	DY475558	Unknown	*Cicer arietinum*	cDNA clone	Experimental	NA
1	3	5	10	NA	Unknown	*Cicer arietinum*	cDNA clone	Experimental	NA
1	3	5	11	EB085052	Unknown	*Cicer arietinum*	cDNA clone	Experimental	NA
1	3	5	12	EB085064	Unknown	*Cicer arietinum*	cDNA clone	Experimental	NA
1	3	5	13	EB085054	Chloroplast DNA	*Cicer arietinum*	cDNA clone	Experimental	NA
1	3	5	14	DY475541	Chloroplast DNA	*Cicer arietinum*	cDNA clone	Experimental	NA
1	3	6	1	DY475531	Unclear	*Cicer arietinum*	cDNA clone	Experimental	NA
1	3	6	2	EB085015	Translational activator	*Cicer arietinum*	cDNA clone	Experimental	NA
1	3	6	3	EB085016	Unknown	*Cicer arietinum*	cDNA clone	Experimental	NA
1	3	6	4	EB085017	Unclear	*Cicer arietinum*	cDNA clone	Experimental	NA
1	3	6	5	NA	Unknown	*Cicer arietinum*	cDNA clone	Experimental	NA
1	3	6	6	NA	Unknown	*Cicer arietinum*	cDNA clone	Experimental	NA
1	3	6	7	DY475556	NADH-plastoquinone oxidoreductase chain 1	*Cicer arietinum*	cDNA clone	Experimental	NA
1	3	6	8	EB085063	Unknown	*Cicer arietinum*	cDNA clone	Experimental	NA
1	3	6	9	NA	Unknown	*Cicer arietinum*	cDNA clone	Experimental	NA
1	3	6	10	EB085018	Acyl-activating enzyme	*Cicer arietinum*	cDNA clone	Experimental	NA
1	3	6	11	EB085034	Unknown	*Cicer arietinum*	cDNA clone	Experimental	NA
1	3	6	12	EB085035	Unknown	*Cicer arietinum*	cDNA clone	Experimental	NA

1	3	6	13	DY475537	Chloroplast Val-tRNA	*Cicer arietinum*	cDNA clone	Experimental	NA
1	3	6	14	DY475555	Chlorophyll a/b binding protein	*Cicer arietinum*	cDNA clone	Experimental	NA
1	3	7	1	DY475351	Unknown	*Cicer arietinum*	cDNA clone	Experimental	NA
1	3	7	2	DY475356	Unknown	*Cicer arietinum*	cDNA clone	Experimental	NA
1	3	7	3	DY475362	Unknown	*Cicer arietinum*	cDNA clone	Experimental	NA
1	3	7	4	DY475364	Unknown	*Cicer arietinum*	cDNA clone	Experimental	NA
1	3	7	5	DY475366	Unknown	*Cicer arietinum*	cDNA clone	Experimental	NA
1	3	7	6	DY475370	Unknown	*Cicer arietinum*	cDNA clone	Experimental	NA
1	3	7	7	DY475437	Unknown	*Cicer arietinum*	cDNA clone	Experimental	NA
1	3	7	8	DY475445	Unknown	*Cicer arietinum*	cDNA clone	Experimental	NA
1	3	7	9	DY475451	Unknown	*Cicer arietinum*	cDNA clone	Experimental	NA
1	3	7	10	DY475461	Unknown	*Cicer arietinum*	cDNA clone	Experimental	NA
1	3	7	11	DY475469	Unknown	*Cicer arietinum*	cDNA clone	Experimental	NA
1	3	7	12	DY475476	Unknown	*Cicer arietinum*	cDNA clone	Experimental	NA
1	3	7	13	DY475482	Unknown	*Cicer arietinum*	cDNA clone	Experimental	NA
1	3	7	14	DY475484	Unknown	*Cicer arietinum*	cDNA clone	Experimental	NA
1	3	8	1	DY475177	Unknown	*Cicer arietinum*	cDNA clone	Experimental	NA
1	3	8	2	DY475185	Unknown	*Cicer arietinum*	cDNA clone	Experimental	NA
1	3	8	3	DY475189	Unknown	*Cicer arietinum*	cDNA clone	Experimental	NA
1	3	8	4	DY475193	Unknown	*Cicer arietinum*	cDNA clone	Experimental	NA
1	3	8	5	DY475263	Unknown	*Cicer arietinum*	cDNA clone	Experimental	NA
1	3	8	6	DY475270	Unknown	*Cicer arietinum*	cDNA clone	Experimental	NA
1	3	8	7	DY475277	Unknown	*Cicer arietinum*	cDNA clone	Experimental	NA
1	3	8	8	DY475280	Unknown	*Cicer arietinum*	cDNA clone	Experimental	NA
1	3	8	9	DY475283	Unknown	*Cicer arietinum*	cDNA clone	Experimental	NA
1	3	8	10	DY475289	Unknown	*Cicer arietinum*	cDNA clone	Experimental	NA
1	3	8	11	DY475293	Unknown	*Cicer arietinum*	cDNA clone	Experimental	NA
1	3	8	12	DY475296	Unknown	*Cicer arietinum*	cDNA clone	Experimental	NA
1	3	8	13	DY475343	Unknown	*Cicer arietinum*	cDNA clone	Experimental	NA
1	3	8	14	DY475349	Unknown	*Cicer arietinum*	cDNA clone	Experimental	NA
1	3	9	1	DY475329	Unclear	*Cicer arietinum*	cDNA clone	Experimental	NA
1	3	9	2	DY475355	Unclear	*Cicer arietinum*	cDNA clone	Experimental	NA
1	3	9	3	DY475515	Unclear	*Cicer arietinum*	cDNA clone	Experimental	NA
1	3	9	4	DY475526	Unclear	*Cicer arietinum*	cDNA clone	Experimental	NA
1	3	9	5	DY475048	Unknown	*Cicer arietinum*	cDNA clone	Experimental	NA
1	3	9	6	DY475055	Unknown	*Cicer arietinum*	cDNA clone	Experimental	NA

1	3	9	7	DY475061	Unknown	*Cicer arietinum*	cDNA clone	Experimental	NA
1	3	9	8	DY475064	Unknown	*Cicer arietinum*	cDNA clone	Experimental	NA
1	3	9	9	DY475075	Unknown	*Cicer arietinum*	cDNA clone	Experimental	NA
1	3	9	10	DY475080	Unknown	*Cicer arietinum*	cDNA clone	Experimental	NA
1	3	9	11	DY475158	Unknown	*Cicer arietinum*	cDNA clone	Experimental	NA
1	3	9	12	DY475160	Unknown	*Cicer arietinum*	cDNA clone	Experimental	NA
1	3	9	13	DY475166	Unknown	*Cicer arietinum*	cDNA clone	Experimental	NA
1	3	9	14	DY475168	Unknown	*Cicer arietinum*	cDNA clone	Experimental	NA
1	3	10	1	DY475239	Membrane sugar-transport protein	*Cicer arietinum*	cDNA clone	Experimental	NA
1	3	10	2	DY475424	Beta adaptin like protein	*Cicer arietinum*	cDNA clone	Experimental	NA
1	3	10	3	DY475468	Cyclic ion channel protein	*Cicer arietinum*	cDNA clone	Experimental	NA
1	3	10	4	DY475512	Aquaporin 2 protein	*Cicer arietinum*	cDNA clone	Experimental	NA
1	3	10	5	DY475053	Unclear	*Cicer arietinum*	cDNA clone	Experimental	NA
1	3	10	6	DY475071	Unclear	*Cicer arietinum*	cDNA clone	Experimental	NA
1	3	10	7	DY475095	Unclear	*Cicer arietinum*	cDNA clone	Experimental	NA
1	3	10	8	DY475099	Unclear	*Cicer arietinum*	cDNA clone	Experimental	NA
1	3	10	9	DY475262	Unclear	*Cicer arietinum*	cDNA clone	Experimental	NA
1	3	10	10	DY475265	Unclear	*Cicer arietinum*	cDNA clone	Experimental	NA
1	3	10	11	DY475273	Unclear	*Cicer arietinum*	cDNA clone	Experimental	NA
1	3	10	12	DY475284	Unclear	*Cicer arietinum*	cDNA clone	Experimental	NA
1	3	10	13	DY475313	Unclear	*Cicer arietinum*	cDNA clone	Experimental	NA
1	3	10	14	DY475322	Unclear	*Cicer arietinum*	cDNA clone	Experimental	NA
1	3	11	1	DY475501	Chloroplast DNA for P700 chlorophyll a-apoproteins	*Cicer arietinum*	cDNA clone	Experimental	NA
1	3	11	2	DY475050	Chloroplast 30S ribosomal protein S3	*Cicer arietinum*	cDNA clone	Experimental	NA
1	3	11	3	DY475073	40S ribosomal protein S3	*Cicer arietinum*	cDNA clone	Experimental	NA
1	3	11	4	DY475110	60S ribosomal protein L17	*Cicer arietinum*	cDNA clone	Experimental	NA
1	3	11	5	DY475120	40S ribosomal protein S18	*Cicer arietinum*	cDNA clone	Experimental	NA
1	3	11	6	DY475123	60S ribosomal protein L10	*Cicer arietinum*	cDNA clone	Experimental	NA
1	3	11	7	DY475421	Acidic 60s ribosomal protein	*Cicer arietinum*	cDNA clone	Experimental	NA
1	3	11	8	DY475429	50S ribosomal protein L7Ae	*Cicer arietinum*	cDNA clone	Experimental	NA
1	3	11	9	DY475479	Serine:glyoxylate aminotransferase (EC 2.6.1.45)/alanine:glyoxylate aminotransferase (EC 2.6.1.44)	*Cicer arietinum*	cDNA clone	Experimental	NA
1	3	11	10	DY475504	S29 ribosomal protein	*Cicer arietinum*	cDNA clone	Experimental	NA
1	3	11	11	DY475507	Anthranilate phosphoribosyltransferase-like protein (EC 2.4.2.18)	*Cicer arietinum*	cDNA clone	Experimental	NA

1	3	11	12	DY475511	Histidine-containing phosphotransferprotein	*Cicer arietinum*	cDNA clone	Experimental	NA
1	3	11	13	DY475087	Mitochondrial 26S rRNA	*Cicer arietinum*	cDNA clone	Experimental	NA
1	3	11	14	DY475544	Chloroplast 4.5S, 5S, 16S and 23S mRNA	*Cicer arietinum*	cDNA clone	Experimental	NA
1	3	12	1	DY475516	Cytochrome C oxidase subunit	*Cicer arietinum*	cDNA clone	Experimental	NA
1	3	12	2	CV793595	Caffeoyl-CoA-Methyltransferase	*Cicer arietinum*	cDNA clone	Experimental	NA
1	3	12	3	CV793589	Homology to an Avr9/Cf9 rapidly elicited protein from *N.tabacum*	*Cicer arietinum*	cDNA clone	Experimental	NA
1	3	12	4	CV793591	S1-3 protein homolog induced by CMV infection in cowpea	*Cicer arietinum*	cDNA clone	Experimental	NA
1	3	12	5	DY475052	Oxygen splitting enhancer protein of photosytem II	*Cicer arietinum*	cDNA clone	Experimental	NA
1	3	12	6	DY475060	Oxygen splitting enhancer protein of photosytem II	*Cicer arietinum*	cDNA clone	Experimental	NA
1	3	12	7	DY475082	ATP synthase (EC 3.6.1.34)	*Cicer arietinum*	cDNA clone	Experimental	NA
1	3	12	8	DY475116	Photosystem II reaction centre I protein	*Cicer arietinum*	cDNA clone	Experimental	NA
1	3	12	9	DY475129	mRNA for light inducible protein precursor of photosystem II	*Cicer arietinum*	cDNA clone	Experimental	NA
1	3	12	10	DY475139	NADH dehydrogenase subunit/NADH-Plastoquinone oxidoreductase subunit	*Cicer arietinum*	cDNA clone	Experimental	NA
1	3	12	11	DY475144	Chloroplast psbB operon	*Cicer arietinum*	cDNA clone	Experimental	NA
1	3	12	12	DY475151	Chlorophyll a/b binding protein	*Cicer arietinum*	cDNA clone	Experimental	NA
1	3	12	13	DY475464	ATP Synthase C chain (lipid binding protein) (EC 3.6.1.34)	*Cicer arietinum*	cDNA clone	Experimental	NA
1	3	12	14	DY475487	Ferredoxin	*Cicer arietinum*	cDNA clone	Experimental	NA
1	3	13	1	DY475384	Serine/threonine protein kinase	*Cicer arietinum*	cDNA clone	Experimental	NA
1	3	13	2	DY475410	Multispanning membrane protein	*Cicer arietinum*	cDNA clone	Experimental	NA
1	3	13	3	DY475170	S-adenosylmethionine decarboxylase (EC 4.1.1.50)	*Cicer arietinum*	cDNA clone	Experimental	NA
1	3	13	4	DY475180	Cytochrome F	*Cicer arietinum*	cDNA clone	Experimental	NA
1	3	13	5	DY475184	Carboxytransferase	*Cicer arietinum*	cDNA clone	Experimental	NA
1	3	13	6	DY475200	Nodulin 21 protein	*Cicer arietinum*	cDNA clone	Experimental	NA
1	3	13	7	DY475213	Carbonic anhydrase (EC 4.2.1.1)	*Cicer arietinum*	cDNA clone	Experimental	NA
1	3	13	8	DY475221	UDP-glucose 4-epimerase (EC 5.1.3.2)	*Cicer arietinum*	cDNA clone	Experimental	NA
1	3	13	9	DY475242	Thiazole biosynthetic enzyme	*Cicer arietinum*	cDNA clone	Experimental	NA
1	3	13	10	DY475282	Trehalose-phosphatase (EC 3.1.3.12)	*Cicer arietinum*	cDNA clone	Experimental	NA
1	3	13	11	DY475449	Cytochrome P450	*Cicer arietinum*	cDNA clone	Experimental	NA
1	3	13	12	DY475458	Cysteine proteinase	*Cicer arietinum*	cDNA clone	Experimental	NA
1	3	13	13	DY475477	Asparagine synthetase (glutamine hydrolysing) (EC 6.3.5.4)	*Cicer arietinum*	cDNA clone	Experimental	NA
1	3	13	14	DY475498	Glucosyltransferase	*Cicer arietinum*	cDNA clone	Experimental	NA
1	3	14	1	DY475227	Myosin heavy-chain protein	*Cicer arietinum*	cDNA clone	Experimental	NA

1	3	14	2	DY475266	DNA binding protein	*Cicer arietinum*	cDNA clone	Experimental	NA
1	3	14	3	DY475357	RNA/ssDNA binding protein	*Cicer arietinum*	cDNA clone	Experimental	NA
1	3	14	4	DY475493	Formyltetrahydrofolate deformylase-like (EC 3.5.1.10)	*Cicer arietinum*	cDNA clone	Experimental	NA
1	3	14	5	DY475070	Dehydration-induced protein	*Cicer arietinum*	cDNA clone	Experimental	NA
1	3	14	6	DY475078	Auxin-repressed protein	*Cicer arietinum*	cDNA clone	Experimental	NA
1	3	14	7	DY475111	Wound-induced protein	*Cicer arietinum*	cDNA clone	Experimental	NA
1	3	14	8	DY475138	Aluminium-induced protein	*Cicer arietinum*	cDNA clone	Experimental	NA
1	3	14	9	DY475517	Farnesylated/isoprenylated protein	*Cicer arietinum*	cDNA clone	Experimental	NA
1	3	14	10	DY475091	Zinc finger protein	*Cicer arietinum*	cDNA clone	Experimental	NA
1	3	14	11	DY475119	Membrane-related protein CP5	*Cicer arietinum*	cDNA clone	Experimental	NA
1	3	14	12	DY475246	GPI-anchored membrane protein	*Cicer arietinum*	cDNA clone	Experimental	NA
1	3	14	13	DY475271	Histidine-rich glycoprotein precursor	*Cicer arietinum*	cDNA clone	Experimental	NA
1	3	14	14	DY475348	Proline-rich structural protein	*Cicer arietinum*	cDNA clone	Experimental	NA
1	4	1	1	NA	NBS-LRR putative RGA Aj516082	*Lens culinaris* (ILL7537)	Genomic PCR product	Experimental	NA
1	4	1	2	NA	NBS-LRR putative RGA Aj516087	*Lens culinaris* (ILL7537)	Genomic PCR product	Experimental	NA
1	4	1	3	NA	NBS-LRR putative RGA Aj516082	*Lens culinaris* (ILL6002)	Genomic PCR product	Experimental	NA
1	4	1	4	NA	NBS-LRR putative RGA Aj516087	*Lens culinaris* (ILL6002)	Genomic PCR product	Experimental	NA
1	4	1	5	NA	NBS-LRR putative RGA Aj516090	*Lens culinaris* (ILL6002)	Genomic PCR product	Experimental	NA
1	4	1	6	NA	Lipoxygenase	*Lens culinaris* (ILL6002)	Genomic PCR product	Experimental	NA
1	4	1	7	NA	NBS-LRR putative RGA LR1	*Lens culinaris* (ILL6002)	Genomic PCR product	Experimental	NA
1	4	1	8	NA	Printing Control	NA	Oligo	Control	Printing
1	4	1	9	NA	Blank	NA	Blank	Control	Negative
1	4	1	10	NA	Blank	NA	Blank	Control	Negative
1	4	2	1	DY396403	Ubiquitin-carboxyl extension	*Lathyrus sativus*	cDNA clone	Experimental	NA
1	4	2	2	DY396401	Ubiquinol-cytochrome C reductase complex 6.7 KDA protein	*Lathyrus sativus*	cDNA clone	Experimental	NA
1	4	2	3	DY396397	Heat shock protein DNAJ homolog	*Lathyrus sativus*	cDNA clone	Experimental	NA
1	4	2	4	DY396268	Histone H2A	*Lathyrus sativus*	cDNA clone	Experimental	NA
1	4	2	5	DY396266	Nucleic acid binding protein-like	*Lathyrus sativus*	cDNA clone	Experimental	NA
1	4	2	6	DY396424	Ubiquitin	*Lathyrus sativus*	cDNA clone	Experimental	NA
1	4	2	7	DY396417	Glycogen synthase kinase-3 homolog MSK-3	*Lathyrus sativus*	cDNA clone	Experimental	NA
1	4	2	8	DY396308	Xyloglucan endotransglycosylase LEXET2	*Lathyrus sativus*	cDNA clone	Experimental	NA
1	4	2	9	NA	NBS-LRR putative RGA Aj516060	*Lens culinaris* (ILL7537)	Genomic PCR product	Experimental	NA
1	4	2	10	NA	NBS-LRR putative RGA Aj516063	*Lens culinaris* (ILL7537)	Genomic PCR product	Experimental	NA
1	4	2	11	NA	NBS-LRR putative RGA Aj516065	*Lens culinaris* (ILL7537)	Genomic PCR product	Experimental	NA
1	4	2	12	NA	NBS-LRR putative RGA Aj516070	*Lens culinaris* (ILL7537)	Genomic PCR product	Experimental	NA

1	4	2	13	NA	NBS-LRR putative RGA Aj516072	*Lens culinaris* (ILL7537)	Genomic PCR product	Experimental	NA
1	4	2	14	NA	NBS-LRR putative RGA Aj516076	*Lens culinaris* (ILL7537)	Genomic PCR product	Experimental	NA
1	4	3	1	DY396415	Caffeoyl-coa O-methyltransferase 4	*Lathyrus sativus*	cDNA clone	Experimental	NA
1	4	3	2	DY396418	Protein transport protein SEC61 gamma subunit	*Lathyrus sativus*	cDNA clone	Experimental	NA
1	4	3	3	DY396425	Disease resistance response protein 230 precursor	*Lathyrus sativus*	cDNA clone	Experimental	NA
1	4	3	4	DY396347	Ripening-related protein	*Lathyrus sativus*	cDNA clone	Experimental	NA
1	4	3	5	DY396429	Putative membrane related protein	*Lathyrus sativus*	cDNA clone	Experimental	NA
1	4	3	6	DY396273	Putative senescence-associated protein	*Lathyrus sativus*	cDNA clone	Experimental	NA
1	4	3	7	DY396309	Transcription initiation protein SPT4 homolog 1	*Lathyrus sativus*	cDNA clone	Experimental	NA
1	4	3	8	DY396344	Ripening-related protein	*Lathyrus sativus*	cDNA clone	Experimental	NA
1	4	3	9	DY396341	Polyubiquitin	*Lathyrus sativus*	cDNA clone	Experimental	NA
1	4	3	10	DY396339	Magnesium chelatase subunit	*Lathyrus sativus*	cDNA clone	Experimental	NA
1	4	3	11	DY396328	Polyubiquitin	*Lathyrus sativus*	cDNA clone	Experimental	NA
1	4	3	12	DY396324	Dehydrin-cognate	*Lathyrus sativus*	cDNA clone	Experimental	NA
1	4	3	13	DY396366	Putative Ubiquitin protein	*Lathyrus sativus*	cDNA clone	Experimental	NA
1	4	3	14	DY396361	Heat shock factor binding protein	*Lathyrus sativus*	cDNA clone	Experimental	NA
1	4	4	1	DY396264	Protein kinase precursor-like	*Lathyrus sativus*	cDNA clone	Experimental	NA
1	4	4	2	DY396269	Putative auxin-repressed protein	*Lathyrus sativus*	cDNA clone	Experimental	NA
1	4	4	3	DY396276	Disease resistance response protein 39 precursor	*Lathyrus sativus*	cDNA clone	Experimental	NA
1	4	4	4	DY396281	Pathogenesis-related protein 4A	*Lathyrus sativus*	cDNA clone	Experimental	NA
1	4	4	5	DY396325	Cutinase negative acting protein	*Lathyrus sativus*	cDNA clone	Experimental	NA
1	4	4	6	DY396332	Lipid transfer protein	*Lathyrus sativus*	cDNA clone	Experimental	NA
1	4	4	7	DY396336	RAC-GTP binding protein-like	*Lathyrus sativus*	cDNA clone	Experimental	NA
1	4	4	8	DY396343	Pathogenesis-related protein	*Lathyrus sativus*	cDNA clone	Experimental	NA
1	4	4	9	DY396350	Nonspecific lipid-transfer protein precursor	*Lathyrus sativus*	cDNA clone	Experimental	NA
1	4	4	10	DY396352	CF-9 resistance gene cluster	*Lathyrus sativus*	cDNA clone	Experimental	NA
1	4	4	11	DY396359	Putative auxin-repressed protein	*Lathyrus sativus*	cDNA clone	Experimental	NA
1	4	4	12	DY396364	ER66 protein/calmodulin binding protein	*Lathyrus sativus*	cDNA clone	Experimental	NA
1	4	4	13	DY396400	EREBP-4	*Lathyrus sativus*	cDNA clone	Experimental	NA
1	4	4	14	DY396407	Defence-related peptide 1	*Lathyrus sativus*	cDNA clone	Experimental	NA
1	4	5	1	EB085036	Chloroplast 30S rRNA	*Cicer arietinum*	cDNA clone	Experimental	NA
1	4	5	2	NA	Unknown	*Cicer arietinum*	cDNA clone	Experimental	NA
1	4	5	3	EB085047	18S rRNA	*Cicer arietinum*	cDNA clone	Experimental	NA
1	4	5	4	NA	Unknown	*Cicer arietinum*	cDNA clone	Experimental	NA
1	4	5	5	EB085048	Unclear	*Cicer arietinum*	cDNA clone	Experimental	NA
1	4	5	6	EB085049	Unknown	*Cicer arietinum*	cDNA clone	Experimental	NA

1	4	5	7	NA	Unknown	*Cicer arietinum*	cDNA clone	Experimental	NA
1	4	5	8	DY475540	26S rRNA	*Cicer arietinum*	cDNA clone	Experimental	NA
1	4	5	9	NA	Unknown	*Cicer arietinum*	cDNA clone	Experimental	NA
1	4	5	10	NA	Unknown	*Cicer arietinum*	cDNA clone	Experimental	NA
1	4	5	11	NA	Normalisation control 3	*Cicer arietinum*	cDNA clone	Control	Normalisation
1	4	5	12	NA	Digested pGEM-T Easy Vector II (Promega) Plasmid *HaeIII*	NA	Digested Plasmid	Control	Negative
1	4	5	13	DY396259	GTP-binding protein SAR1A	*Lathyrus sativus*	cDNA clone	Experimental	NA
1	4	5	14	DY396261	Receptor-like protein kinase	*Lathyrus sativus*	cDNA clone	Experimental	NA
1	4	6	1	NA	Unknown	*Cicer arietinum*	cDNA clone	Experimental	NA
1	4	6	2	NA	Unknown	*Cicer arietinum*	cDNA clone	Experimental	NA
1	4	6	3	NA	Unknown	*Cicer arietinum*	cDNA clone	Experimental	NA
1	4	6	4	EB085031	Cytochrome P450	*Cicer arietinum*	cDNA clone	Experimental	NA
1	4	6	5	DY475557	18S rRNA, partial	*Cicer arietinum*	cDNA clone	Experimental	NA
1	4	6	6	NA	Unknown	*Cicer arietinum*	cDNA clone	Experimental	NA
1	4	6	7	DY475535	Unknown	*Cicer arietinum*	cDNA clone	Experimental	NA
1	4	6	8	EB085033	5.8S, 18S and 25S rRNA	*Cicer arietinum*	cDNA clone	Experimental	NA
1	4	6	9	EB085024	Unknown	*Cicer arietinum*	cDNA clone	Experimental	NA
1	4	6	10	DY475534	Chlorophyll a/b binding protein	*Cicer arietinum*	cDNA clone	Experimental	NA
1	4	6	11	EB085025	Unknown	*Cicer arietinum*	cDNA clone	Experimental	NA
1	4	6	12	EB085062	Unknown	*Cicer arietinum*	cDNA clone	Experimental	NA
1	4	6	13	NA	Unknown	*Cicer arietinum*	cDNA clone	Experimental	NA
1	4	6	14	NA	Unknown	*Cicer arietinum*	cDNA clone	Experimental	NA
1	4	7	1	DY475392	Unknown	*Cicer arietinum*	cDNA clone	Experimental	NA
1	4	7	2	DY475401	Unknown	*Cicer arietinum*	cDNA clone	Experimental	NA
1	4	7	3	DY475412	Unknown	*Cicer arietinum*	cDNA clone	Experimental	NA
1	4	7	4	DY475416	Unknown	*Cicer arietinum*	cDNA clone	Experimental	NA
1	4	7	5	DY475428	Unknown	*Cicer arietinum*	cDNA clone	Experimental	NA
1	4	7	6	DY475432	Unknown	*Cicer arietinum*	cDNA clone	Experimental	NA
1	4	7	7	DY475490	Unknown	*Cicer arietinum*	cDNA clone	Experimental	NA
1	4	7	8	DY475503	Unknown	*Cicer arietinum*	cDNA clone	Experimental	NA
1	4	7	9	DY475513	Unknown	*Cicer arietinum*	cDNA clone	Experimental	NA
1	4	7	10	DY475520	Unknown	*Cicer arietinum*	cDNA clone	Experimental	NA
1	4	7	11	DY475529	Unknown	*Cicer arietinum*	cDNA clone	Experimental	NA
1	4	7	12	EB085013	26S rRNA	*Cicer arietinum*	cDNA clone	Experimental	NA
1	4	7	13	NA	Unknown	*Cicer arietinum*	cDNA clone	Experimental	NA
1	4	7	14	NA	Unknown	*Cicer arietinum*	cDNA clone	Experimental	NA

1	4	8	1	DY475232	Unknown	*Cicer arietinum*	cDNA clone	Experimental	NA
1	4	8	2	DY475241	Unknown	*Cicer arietinum*	cDNA clone	Experimental	NA
1	4	8	3	DY475253	Unknown	*Cicer arietinum*	cDNA clone	Experimental	NA
1	4	8	4	DY475256	Unknown	*Cicer arietinum*	cDNA clone	Experimental	NA
1	4	8	5	DY475299	Unknown	*Cicer arietinum*	cDNA clone	Experimental	NA
1	4	8	6	DY475310	Unknown	*Cicer arietinum*	cDNA clone	Experimental	NA
1	4	8	7	DY475314	Unknown	*Cicer arietinum*	cDNA clone	Experimental	NA
1	4	8	8	DY475326	Unknown	*Cicer arietinum*	cDNA clone	Experimental	NA
1	4	8	9	DY475330	Unknown	*Cicer arietinum*	cDNA clone	Experimental	NA
1	4	8	10	DY475336	Unknown	*Cicer arietinum*	cDNA clone	Experimental	NA
1	4	8	11	DY475338	Unknown	*Cicer arietinum*	cDNA clone	Experimental	NA
1	4	8	12	DY475340	Unknown	*Cicer arietinum*	cDNA clone	Experimental	NA
1	4	8	13	DY475377	Unknown	*Cicer arietinum*	cDNA clone	Experimental	NA
1	4	8	14	DY475390	Unknown	*Cicer arietinum*	cDNA clone	Experimental	NA
1	4	9	1	DY475448	Unclear	*Cicer arietinum*	cDNA clone	Experimental	NA
1	4	9	2	DY475495	Unclear	*Cicer arietinum*	cDNA clone	Experimental	NA
1	4	9	3	DY475084	Unknown	*Cicer arietinum*	cDNA clone	Experimental	NA
1	4	9	4	DY475089	Unknown	*Cicer arietinum*	cDNA clone	Experimental	NA
1	4	9	5	DY475098	Unknown	*Cicer arietinum*	cDNA clone	Experimental	NA
1	4	9	6	DY475102	Unknown	*Cicer arietinum*	cDNA clone	Experimental	NA
1	4	9	7	DY475115	Unknown	*Cicer arietinum*	cDNA clone	Experimental	NA
1	4	9	8	DY475130	Unknown	*Cicer arietinum*	cDNA clone	Experimental	NA
1	4	9	9	DY475143	Unknown	*Cicer arietinum*	cDNA clone	Experimental	NA
1	4	9	10	DY475156	Unknown	*Cicer arietinum*	cDNA clone	Experimental	NA
1	4	9	11	DY475206	Unknown	*Cicer arietinum*	cDNA clone	Experimental	NA
1	4	9	12	DY475210	Unknown	*Cicer arietinum*	cDNA clone	Experimental	NA
1	4	9	13	DY475216	Unknown	*Cicer arietinum*	cDNA clone	Experimental	NA
1	4	9	14	DY475223	Unknown	*Cicer arietinum*	cDNA clone	Experimental	NA
1	4	10	1	DY475118	Unclear	*Cicer arietinum*	cDNA clone	Experimental	NA
1	4	10	2	DY475173	Unclear	*Cicer arietinum*	cDNA clone	Experimental	NA
1	4	10	3	DY475186	Unclear	*Cicer arietinum*	cDNA clone	Experimental	NA
1	4	10	4	DY475214	Unclear	*Cicer arietinum*	cDNA clone	Experimental	NA
1	4	10	5	DY475218	Unclear	*Cicer arietinum*	cDNA clone	Experimental	NA
1	4	10	6	DY475546	Unclear	*Cicer arietinum*	cDNA clone	Experimental	NA
1	4	10	7	DY475233	Unclear	*Cicer arietinum*	cDNA clone	Experimental	NA
1	4	10	8	DY475257	Unclear	*Cicer arietinum*	cDNA clone	Experimental	NA

1	4	10	9	DY475376	Unclear	*Cicer arietinum*	cDNA clone	Experimental	NA
1	4	10	10	DY475386	Unclear	*Cicer arietinum*	cDNA clone	Experimental	NA
1	4	10	11	DY475389	Unclear	*Cicer arietinum*	cDNA clone	Experimental	NA
1	4	10	12	DY475400	Unclear	*Cicer arietinum*	cDNA clone	Experimental	NA
1	4	10	13	DY475411	Unclear	*Cicer arietinum*	cDNA clone	Experimental	NA
1	4	10	14	DY475438	Unclear	*Cicer arietinum*	cDNA clone	Experimental	NA
1	4	11	1	DY475317	40S ribosomal protein S8	*Cicer arietinum*	cDNA clone	Experimental	NA
1	4	11	2	DY475334	Chloroplast 30S ribosomal protein S7	*Cicer arietinum*	cDNA clone	Experimental	NA
1	4	11	3	DY475346	Elongation factor (translation initiation factor)	*Cicer arietinum*	cDNA clone	Experimental	NA
1	4	11	4	DY475359	50S ribosomal protein L27	*Cicer arietinum*	cDNA clone	Experimental	NA
1	4	11	5	DY475394	60S ribosomal protein L39	*Cicer arietinum*	cDNA clone	Experimental	NA
1	4	11	6	DY475406	FKBP-type peptidyl-prolyl cis-trans isomerase (EC 5.2.1.8)	*Cicer arietinum*	cDNA clone	Experimental	NA
1	4	11	7	DY475545	Chloroplast 4.5S, 5S, 16S and 23S mRNA	*Cicer arietinum*	cDNA clone	Experimental	NA
1	4	11	8	DY475150	18S nuclear rRNA	*Cicer arietinum*	cDNA clone	Experimental	NA
1	4	11	9	DY475154	Chloroplast 4.5S/5S/16S/23S mRNA	*Cicer arietinum*	cDNA clone	Experimental	NA
1	4	11	10	DY475211	26S rRNA	*Cicer arietinum*	cDNA clone	Experimental	NA
1	4	11	11	DY475375	Sucrose responsive transcription factor	*Cicer arietinum*	cDNA clone	Experimental	NA
1	4	11	12	DY475059	Nuclear transport factor	*Cicer arietinum*	cDNA clone	Experimental	NA
1	4	11	13	DY475124	Aquaporin	*Cicer arietinum*	cDNA clone	Experimental	NA
1	4	11	14	DY475174	Aquaporin membrane protein	*Cicer arietinum*	cDNA clone	Experimental	NA
1	4	12	1	CV793607	Flavonol glucosyl transferase	*Cicer arietinum*	cDNA clone	Experimental	NA
1	4	12	2	CV793609	Similar to elicitor-inducible receptor-like protein	*Cicer arietinum*	cDNA clone	Experimental	NA
1	4	12	3	CV793590	Protein translation factor homolog (translation initiation factor nps45)	*Cicer arietinum*	cDNA clone	Experimental	NA
1	4	12	4	CV793588	Gamma-thionen type defensin/protease inhibitor	*Cicer arietinum*	cDNA clone	Experimental	NA
1	4	12	5	DY475176	Chloroplast genome DNA	*Cicer arietinum*	cDNA clone	Experimental	NA
1	4	12	6	DY475224	Plastocyanin	*Cicer arietinum*	cDNA clone	Experimental	NA
1	4	12	7	DY475285	Photosystem I reaction centre subunit VI-2	*Cicer arietinum*	cDNA clone	Experimental	NA
1	4	12	8	DY475294	NADH-ubiquinone oxidoreductase (EC 1.6.5.3)	*Cicer arietinum*	cDNA clone	Experimental	NA
1	4	12	9	DY475305	Thylakoid protein	*Cicer arietinum*	cDNA clone	Experimental	NA
1	4	12	10	DY475345	Photosystem I assembly protein ycf3	*Cicer arietinum*	cDNA clone	Experimental	NA
1	4	12	11	DY475423	ATP synthase (EC 3.6.1.34)	*Cicer arietinum*	cDNA clone	Experimental	NA
1	4	12	12	DY475434	Proton pump interactor protein	*Cicer arietinum*	cDNA clone	Experimental	NA
1	4	12	13	DY475201	60S ribosomal protein L34	*Cicer arietinum*	cDNA clone	Experimental	NA
1	4	12	14	DY475258	40S ribosomal protein S11	*Cicer arietinum*	cDNA clone	Experimental	NA
1	4	13	1	DY475141	Beta-galactosidase (EC 3.2.1.23)	*Cicer arietinum*	cDNA clone	Experimental	NA

1	4	13	2	DY475152	Cytidine deaminase enzyme	*Cicer arietinum*	cDNA clone	Experimental	NA
1	4	13	3	DY475302	4-alpha-glucanotransferase (EC 2.4.1.25)	*Cicer arietinum*	cDNA clone	Experimental	NA
1	4	13	4	DY475308	Glutamate dehydrogenase (EC 1.4.1.3)	*Cicer arietinum*	cDNA clone	Experimental	NA
1	4	13	5	DY475321	Mitochondrial glyoxylase	*Cicer arietinum*	cDNA clone	Experimental	NA
1	4	13	6	DY475548	Cytosolic fructose 1,6-bisphosphatase (EC 3.1.3.11)	*Cicer arietinum*	cDNA clone	Experimental	NA
1	4	13	7	DY475393	Cytochrome C biogenesis protein ccsA	*Cicer arietinum*	cDNA clone	Experimental	NA
1	4	13	8	DY475398	Glutamine synthetase (glutamate ammonia ligase) (EC 6.3.1.2)	*Cicer arietinum*	cDNA clone	Experimental	NA
1	4	13	9	DY475408	Xylosidase	*Cicer arietinum*	cDNA clone	Experimental	NA
1	4	13	10	DY475417	Probable 3-hydroxyisobutyrate dehydrogenase (HIBADH) mitochondrial precursor (EC 1.1.1.31)	*Cicer arietinum*	cDNA clone	Experimental	NA
1	4	13	11	CV793597	Pathogenesis-related protein 4A	*Cicer arietinum*	cDNA clone	Experimental	NA
1	4	13	12	CV793599	Protein containing leucine-zipper motif	*Cicer arietinum*	cDNA clone	Experimental	NA
1	4	13	13	CV793601	Leucine-zipper containing protein	*Cicer arietinum*	cDNA clone	Experimental	NA
1	4	13	14	CV793605	Multi-resistance protein ABC transporter	*Cicer arietinum*	cDNA clone	Experimental	NA
1	4	14	1	DY475190	S-adenosylmethionine synthetase enzyme (EC 2.5.1.6)	*Cicer arietinum*	cDNA clone	Experimental	NA
1	4	14	2	DY475207	Endoxyloglucan transferase involved in water-stress	*Cicer arietinum*	cDNA clone	Experimental	NA
1	4	14	3	DY475225	Proline oxidase	*Cicer arietinum*	cDNA clone	Experimental	NA
1	4	14	4	DY475250	Glutathione S-transferase (EC 2.5.1.18)	*Cicer arietinum*	cDNA clone	Experimental	NA
1	4	14	5	DY475276	Homocysteine methyltransferase	*Cicer arietinum*	cDNA clone	Experimental	NA
1	4	14	6	DY475328	Ubiquitin conjugating protein	*Cicer arietinum*	cDNA clone	Experimental	NA
1	4	14	7	DY475397	Superoxide dismutase copper chaperone precursor	*Cicer arietinum*	cDNA clone	Experimental	NA
1	4	14	8	DY475474	Heat shock protein	*Cicer arietinum*	cDNA clone	Experimental	NA
1	4	14	9	DY475470	Protein kinase mRNA	*Cicer arietinum*	cDNA clone	Experimental	NA
1	4	14	10	DY475508	Hypothetical protein with a membrane spanning ring-H2 finger domain	*Cicer arietinum*	cDNA clone	Experimental	NA
1	4	14	11	DY475066	Cysteine proteinase	*Cicer arietinum*	cDNA clone	Experimental	NA
1	4	14	12	DY475096	Glyceraldehyde 3-phosphate dehydrogenase (EC 1.2.1.12)	*Cicer arietinum*	cDNA clone	Experimental	NA
1	4	14	13	DY475543	Fructose-1,6-bisphosphatase (EC 3.1.3.11)	*Cicer arietinum*	cDNA clone	Experimental	NA
1	4	14	14	DY475113	Cytochrome C oxidase subunit	*Cicer arietinum*	cDNA clone	Experimental	NA
